# Toward Integrated Large-Scale Environmental Monitoring Using WSN/UAV/Crowdsensing: A Review of Applications, Signal Processing, and Future Perspectives

**DOI:** 10.3390/s22051824

**Published:** 2022-02-25

**Authors:** Alessio Fascista

**Affiliations:** Department of Engineering, University of Salento, Via Monteroni, 73100 Lecce, Italy; alessio.fascista@unisalento.it

**Keywords:** environmental monitoring, wireless sensor networks (WSNs), unmanned aerial vehicles (UAVs), crowdsensing, signal processing, pollution monitoring, natural disasters

## Abstract

Fighting Earth’s degradation and safeguarding the environment are subjects of topical interest and sources of hot debate in today’s society. According to the United Nations, there is a compelling need to take immediate actions worldwide and to implement large-scale monitoring policies aimed at counteracting the unprecedented levels of air, land, and water pollution. This requires going beyond the legacy technologies currently employed by government authorities and adopting more advanced systems that guarantee a continuous and pervasive monitoring of the environment in all its different aspects. In this paper, we take the research on integrated and large-scale environmental monitoring a step further by providing a comprehensive review that covers transversally all the main applications of wireless sensor networks (WSNs), unmanned aerial vehicles (UAVs), and crowdsensing monitoring technologies. By outlining the available solutions and current limitations, we identify in the cooperation among terrestrial (WSN/crowdsensing) and aerial (UAVs) sensing, coupled with the adoption of advanced signal processing techniques, the major pillars at the basis of future integrated (air, land, and water) and large-scale environmental monitoring systems. This review not only consolidates the progresses achieved in the field of environmental monitoring, but also sheds new lights on potential future research directions and synergies among different research areas.

## 1. Introduction

Preserving and protecting the environment is, today more than ever, an imperative requirement for modern society. Unmanageable levels of pollution, unpredictable climate changes, and over-exploitation of natural resources are severely harming human health and the general well-being of society while at the same time hindering a sustainable growth of the global economy. According to the Sixth Intergovernmental Panel on Climate Change (IPCC) report released by United Nations in 2021 [1], human activities have caused an average increase in global temperatures of about 1.1${}^{\circ }$ C compared to the period before the industrial revolution: global warming has not only increased the frequency and intensity of disastrous environmental phenomena such as wildfires, toxic rains, and floodings, but has also damaged and aggravated the situation of ecosystems worldwide. Notably, the uncontrolled greenhouse gases generated from the increasing urbanization and industrialization, agricultural imbalances, and aggressive de-forestation are at the basis of dramatic human diseases such as asthma, lung cancer, chronic pulmonary disease, and pneumonia, causing more than 7 million premature deaths per year [2,3]. To counteract such unprecedented issues, it becomes necessary to exploit all the commercially available technologies to guarantee a continuous and pervasive monitoring of the environment in all its different aspects (air, land, and water). In view of the continually growing sources of pollution and natural hazards, monitoring systems should be able to dynamically adapt to different contexts, to manage a huge amount of heterogeneous environmental data, and to operate over large geographical scales. This requires rethinking the way such systems have been designed so far, following a new paradigm in which environmental monitoring is not merely intended as a passive collection of environmental data in different contexts, to be used for detecting possible breaches of safety-critical thresholds, but involves more advanced processes that aim at extracting more accurate and complete knowledge about the monitored phenomena, possibly in real-time, to devise both *proactive* and *reactive* strategies able to limit the environmental damages and predict their potential impacts.

More traditional monitoring systems currently adopted by government authorities consist of a few fixed stations, equipped with advanced sensors and measurement units, which are sparsely deployed over large geographical areas. Practical examples include the meteorological monitoring stations [4], the diffused seismographs systems for earthquake detection [5], or the oceanic report systems [6], just to name a few. To complement such terrestrial systems, observations from satellite and airborne platforms have been largely considered, not only for strict monitoring purposes [7,8,9,10], but also for building accurate 3D models of the earth’s surface [11]. Despite the quite high precision provided through their dedicated equipment, such systems are tailored for single or limited types of environmental analyses and can provide observations of the physical phenomena only at a very small number of locations. For the specific cases of satellite and airborne systems, the rate of data acquisition can be as low as a few observations per day, and the measurement accuracy is severely impaired in the presence of bad weather conditions (clouds, fog, rain). Unfortunately, most of the main environmental indicators (e.g., air quality, temperature, pressure, water turbidity) has the intrinsic characteristic of experiencing rapid changes even at distances in the order of a few meters, especially in highly dynamic environments such as urban areas [12]. Chiefly, the cost and time required to install and periodically maintain the sophisticated hardware and software components is often unaffordable [13]. In this respect, a paradigm shift is necessary where pervasive and fine-grained environmental monitoring is performed by means of a larger number of low-cost sensing units which, by providing a more capillary coverage of the target areas and an increased sensing rate, are able to correctly capture the spatio-temporal variations of the physical phenomena of interest.

A first step in this direction is represented by the adoption of terrestrial wireless sensor networks (WSNs). WSNs applied to monitoring contexts are a practical example of the emerging internet-of-things (IoT) paradigm, where hundreds or thousands of interconnected tiny devices are collaboratively used to observe and react according to a given phenomenon in the surrounding environment [14]. Fostered by the rapid advances in the field of sensor miniaturization, microelectronics, and low-power wireless communications, WSN nodes are realized as smart and compact devices equipped with a number of inexpensive sensors measuring different environmental parameters such as particulate matters, temperature, pressure, pH level, water conductivity, among many others [15]. Compared to the above-discussed traditional monitoring systems, a strategic deployment of a WSN offers a substantial opportunity to obtain a more accurate knowledge of environmental phenomena by leveraging the finer sampling capabilities of the sensing network. Although WSNs have been transversally applied to different environmental contexts such as air pollution monitoring [16], wildfire early detection [17], and water monitoring [18], this technology is not without significant limitations. Indeed, WSNs are typically installed at fixed and static locations and can provide a sufficient coverage only over very small target areas. Moreover, WSN nodes are characterized by a quite limited autonomy, a short communication range, and reduced processing and storage resources.

Over the last two decades, there has been a growing interest in the adoption of unmanned aerial vehicles (UAVs), also known as drones, for environmental monitoring purposes. Thanks to their aerial inspection capabilities, UAVs can reach remote and hardly accessible locations and exploit their flexible flying characteristics to perform monitoring operations at different spatial resolutions (namely, different altitudes and view angles) while guaranteeing much higher sampling rates. The ongoing downsizing of integrated sensor technologies allows UAV platforms to be endorsed with a multitude of different sensors, ranging from common optical (RGB) cameras to more advanced multispectral/hyperspectral and LIDAR sensors. By capturing detailed environmental data over different spectral ranges, UAVs promoted the design of new approaches to reveal the physical characteristics of materials dispersed in a monitored site, discriminating between natural and pollutant materials and reconstructing accurate 2D/3D maps of the land surfaces even in areas where other airborne or spaceborne technologies are not applicable [19,20]. Furthermore, UAVs proved to be valuable tools for supporting public authorities in managing all the phases of a natural disaster (e.g., wildfire, flood, earthquake), from prevention and preliminary assessment of the damages to the final recovery [21,22]. There are, however, some open issues, including the potential safety threats related to the use of UAVs [23,24,25], leading to restrictions on their flight operations, the correct calibration of mounted sensors [26], and the need for accurate localization and spatial contextualization of the collected data [27], which should be still faced before considering UAVs as a fully matured technology for environmental monitoring.

Nowadays, the capillary diffusion of smartphones and wearable devices (smart watches, smart wristbands) along with the rich set of built-in and Bluetooth sensors (e.g., antennas, microphones, cameras) are the key enablers driving the successful application of the crowdsensing paradigm to the field of environmental monitoring. Crowdsensing relies on the idea of exploiting the sensing and communication capabilities embedded in daily used mobile devices to opportunistically collect, process, and store environmental data at practically *zero-cost* [28]. Stimulated by the possibility of actively contributing to environmental protection, citizens can be personally involved in the sensing campaign and additionally provide their subjective perceptions about the environment, further enriching the information obtained by a mere sampling of environmental data [29]. Besides people, also the most common private/public transportation systems such as cars, buses, taxis, bicycles, and trains represent valuable crowdsensing platforms, which can carry sensors for different pollution monitoring (e.g., air pollution, acoustic noise pollution) and exploit their mobility to cover larger areas [30]. With the forthcoming vehicle-to-anything (V2X) communication technologies, smart vehicles can also collaborate among each other, further increasing the potential of crowdsensing in the emerging contexts of smart cities and intelligent transportation systems (ITSs) [31,32]. Evidence shows that even with a small set of recruited crowdsensing devices, it is possible to identify areas with systematically higher levels of pollution. The success of crowdsensing is strongly related to the willingness of citizens to collaborate by making their private smartphones or vehicles available for the sensing campaigns. Unfortunately, selfish and privacy concerns still hamper the wide diffusion of such a paradigm [33].

The intrinsic multidisciplinarity of the environmental monitoring problem has led to a plethora of different methodologies and algorithms available in the literature, scattered across a number of different research communities. Considerable research efforts have been made to improve the monitoring capabilities of each individual technology (WSN/UAV/crowdsensing) and, recently, the possible combinations of multiple technologies have started to be investigated. Despite the evident advances achieved with respect to legacy monitoring systems, there still exist significant technological and methodological gaps to be filled in order to ensure an *integrated monitoring* of the environment in all its aspects (air, land, and water) while offering a cost-effective and scalable solution to support a coverage over a *large geographical scale*. More specifically, the considerable complexity is mainly related to the great diversity among the air, land, and marine contexts, to the huge heterogeneity of environmental data that need to be jointly processed, and more generally to the specific characteristics of each monitoring task.

Different survey papers have been already proposed in the literature, mainly focused on each individual aerial (UAV) or terrestrial (WSN/crowdsensing) technology and its applications in environmental monitoring, as summarized in Table 1. Specifically, a number of reviews have been devoted to the use of WSNs, highlighting their benefits compared to legacy monitoring systems [34] and their role in different contexts such as marine monitoring [35], water quality assessment [36], air pollution monitoring [37], urban noise monitoring [38], and precision agriculture [39]. Several surveys also investigated the sensing capabilities of UAV platforms by considering the available hardware/software technologies [40] and the current practices related to their use [41] and studying their application in agriculture and forestry monitoring [42], coastal habitat mapping [43], early detection of forest fires [44], landslide monitoring [45], and air quality assessment [46]. The shift toward the use of mobile crowdsensing as a viable and cost-effective data collection paradigm has been thoroughly discussed in [47] and further investigated in [48], where the emerging technologies of wearable sensors have been classified and characterized in detail. A rather complete overview of the potential uses of mobile crowdsensing in smart agriculture was provided in [49], while [50] illustrated the main applications of smartphone-based environmental monitoring in the rising IoT era. In addition, a comprehensive analysis on the use of social media as a promising crowdsensing tool for natural-disaster management has been conducted in [51]. Recently, some review papers started to appear that consider collaborative monitoring systems based on a combination of two technologies—for instance, WSN-UAV [52,53] and WSN-crowdsensing [54]—but with the main purposes of improving specific tasks such as the management of natural disasters or the monitoring of pollution in urban areas, without, however, taking into account all the remaining aspects involved in environmental monitoring.

In this respect, the general scope of this paper is to provide a comprehensive review that transversally considers the three sensing technologies (WSN, UAV, and crowdsensing) and combines their benefits in a synergistic manner, taking into account all the different application contexts at hand (air, land, and water) and jointly considering the main involved tasks, from data acquisition to communication and processing. The review not only simplifies the understanding of the current solutions and available techniques, but also sheds new light on the major ingredients that should be considered to design future *integrated* and *large-scale* environmental monitoring systems. Specifically, the main contributions of the paper are as follows:

(i) An in-depth review of the main applications of each individual technology (WSN, UAV, and crowdsensing) to environmental monitoring is conducted, classifying the existing solutions based on their specific fields of application: (a) air monitoring, (b) land monitoring, and (c) water/marine monitoring. Based on such a classification, the main benefits and current limitations of each technology are then outlined.
(ii) A detailed overview of the signal processing techniques applied in the field of environmental monitoring is presented, showing how they provide elegant and efficient solutions to many pivotal aspects of monitoring tasks, from the optimal deployment of sensing nodes to the accurate modeling and reconstruction of the physical phenomena of interest.
(iii) The main components of a high-level architecture that leverages the different air–ground sensing capabilities of WSNs, UAVs, and crowdsensing, to enable an integrated and large-scale monitoring of the environment, are identified. The architecture includes all application scenarios (air, land, and water) and interprets the whole ecosystem (WSN/UAV/crowdsensing) as a unified multi-agent and multi-system framework, using advanced signal processing for low cost and scalability.
(iv) Promising future research directions and synergies between different research areas envisioned as key enablers for integrated large-scale environmental monitoring are finally discussed.

**Figure 1:**
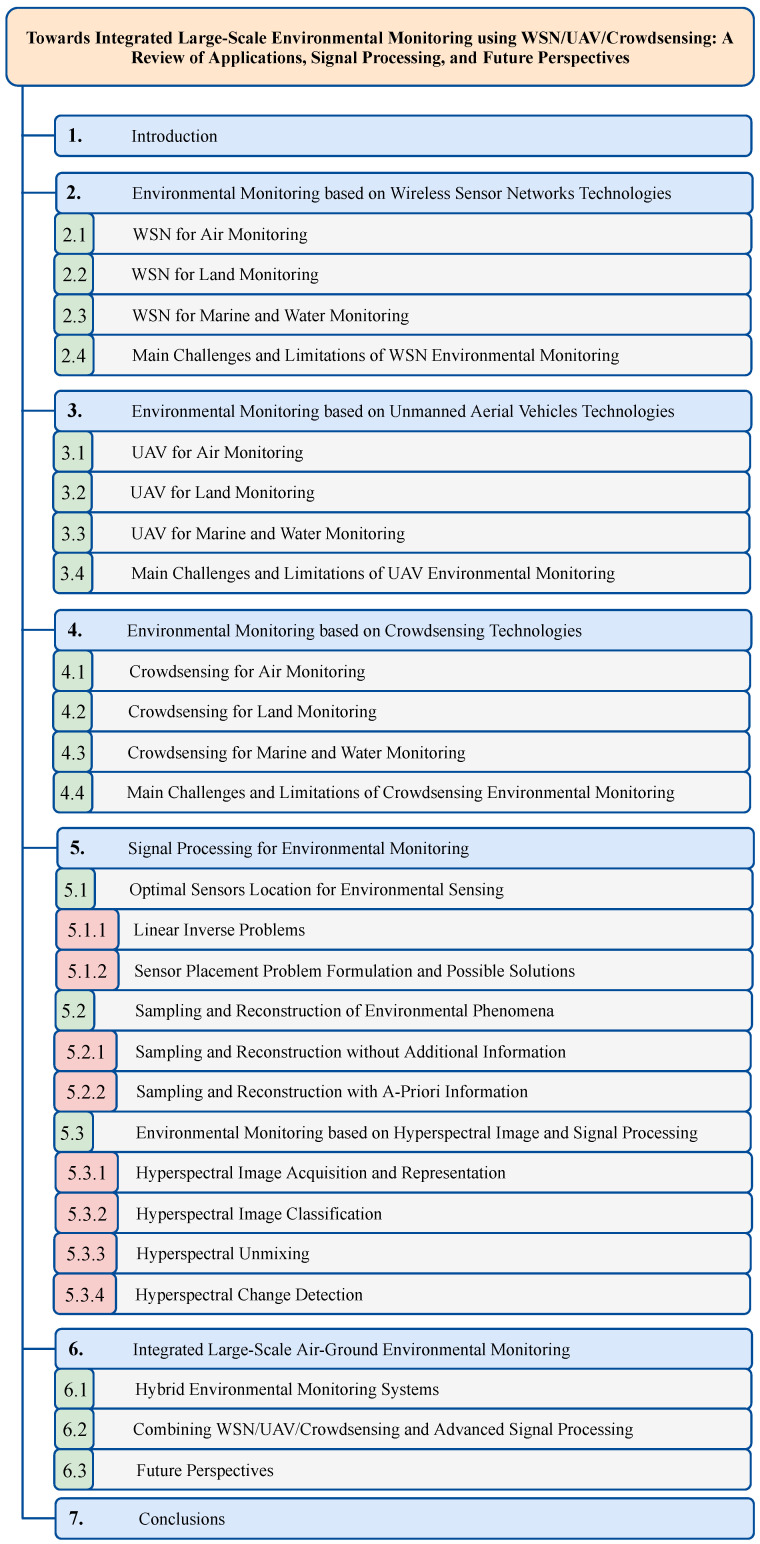
Structure and organization of the review.

The structure and organization of the paper is depicted in Figure 1.

## 2. Environmental Monitoring Based on Wireless Sensor Network Technologies

**Figure 2:**
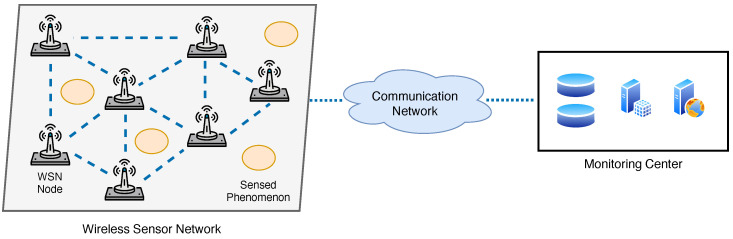
General architecture of an environmental monitoring system based on a WSN.

The general architecture of an environmental monitoring system based on a terrestrial WSN consists of hundreds or thousands of low-cost sensor nodes located in strategic points of the monitored site (whose position is assumed to be known) and of one or more monitoring centers responsible for collecting and processing all the data acquired by the sensor nodes, as illustrated in Figure 2.

Each sensor node in the WSN is a low-power device equipped with:

- A *microprocessor unit* to control and manage the local tasks and to perform basic computations on the acquired data.
- An *internal memory* with limited capacity used to store small batches of collected data before transferring them to the monitoring centers.
- A *transceiver* for establishing communication links with the other nodes in the network and with the monitoring centers.
- A *sensing unit* equipped with several dedicated sensors (e.g., chemical, thermal, biological) to measure and monitor the environmental parameters of interest.

WSN nodes can collect different types of environmental data in different formats and resolutions, e.g., analog or digital, static or dynamic, spatial or temporal, and images or video sequences, just to name a few. At the monitoring center, the high volume of collected data is properly stored in dedicated databases and processed through high-performance computing systems. Data are typically pre-processed to remove possible outliers before being analyzed and visualized using, e.g., common Geographic Information Systems (GISs), in combination with additional information from satellites (maps) and, possibily, coupled with the predictions produced by spatial–temporal models of the pollutants. The results of the analyses are subsequently made available through specific web platforms to guarantee seamless access to all the authorized entities (private and/or public).

Compared to more traditional systems based on few fixed monitoring stations, WSNs revolutionize the sensing task by enabling an accurate, pervasive, and real-time monitoring of the main environmental processes and parameters thanks to the increased spatial resolution and differentiated sensing capacity of the network. Commercial off-the-shelf (COTS) sensors mounted on the sensing unit of WSN nodes can measure a number of physical parameters such as temperature, humidity, and pressure, as well as some of the most important chemical pollutants. In Table 2, we report the most common type of sensors used in WSN-based environmental monitoring systems, including their operating range and manufacturing technology. For more comprehensive discussions about the various environmental sensors, we refer the interested reader to the dedicated surveys [55,56,57,58,59,60]. In the following, we provide a review of some representative approaches proposed in the domain of WSN-based environmental monitoring, classified based on their fields of application (air, land, or sea). On the basis of the reviewed literature, we then conclude the section by highlighting the common challenges and the main limitations of WSN-based monitoring systems.

### 2.1. WSN for Air Monitoring

WSN technologies have received significant attention in the area of air environmental monitoring. With the progressive evolution toward the emerging reality of smart cities, monitoring the quality of the air in all its aspects (chemical, electromagnetic, and acoustic noise pollution) in crowded urban areas is becoming a major concern. Indeed, the highly dynamic nature of such environments triggers frequent changes in the concentrations of the air pollutants, which in the worst cases may occur in the scale of few seconds over time and of few meters in space [61]. Enabling real-time air monitoring in these harsh environments through a scalable, reprogrammable and low-cost WSN deployed over traffic/street lights is the objective of some important projects such as [62,63,64]. Another important aspect of WSN-based air monitoring concerns the quite high power and response time required by greenhouse gases sensors (CO, CO${}_{2}$, SO${}_{x}$, NO${}_{x}$, O${}_{2}$), especially those based on MOX technology. In [65,66,67,68], such issues are explicitly taken into account at the design stage and solved using a combination of power reduction techniques that operate at both sensors and network levels, also including some context-adaptive strategies [69]. Minimal invasiveness is another desired property when designing and deploying effective WSN-based air monitoring systems, especially for indoor environments. In [70,71,72,73], different multilayer architectures are presented that enable a distributed monitoring of the air environmental parameters using only a limited number of deployed sensors while still preserving the low-cost and flexible characteristics of a WSN. The optimal deployment of WSN nodes for finer spatio-temporal air monitoring is addressed in [74,75,76]. The optimization problem is formulated by explicitly taking into account the dynamic diffusion of the air pollutants, represented by means of atmospheric dispersion models, including also some realistic connectivity issues among the nodes in the WSN. Preliminary experiments on real datasets indicate that the actual sensing capabilities of the WSN are strongly affected by the weather conditions, with the estimation performance that tends to increase when the sensors are deployed at an altitude close to the main concentrations of the air pollutants. Moreover, the deployment costs, which are quantified as the total number of WSN nodes required to guarantee a predefined error-bounded coverage, can be progressively lowered as the number of pollutant sources or the number of emissions from a single source increases.

WSN technologies have also proven their validity in monitoring different sources of environmental acoustic noise (ranging from those generated by common transportation systems to industrial manufacturing and building construction) that can be found in most human-centric areas [38,77,78]. Compared to sensing chemical air pollutants, which involves the processing of separate data from each dedicated sensor, acoustic noise monitoring introduces additional challenges related to the correct separation, classification, and identification of the several noise contributions that are superimposed at the receiving microphones [79]. In this respect, a lot of work has been devoted to the design of advanced acoustic signal processing techniques that elaborate on well-known methods such as beamforming [80], source localization [81], array calibration [82], and noise reduction [83]. An evident trade-off between accuracy and deployment costs tends to emerge from the reviewed literature: on the one hand, approaches such as [84,85,86] consider class-A acoustic sensors to build near real-time maps that provide a detailed analysis of the acoustic environment, including the classification of all the diverse sources of noise. Such approaches are however limited to very small areas (WSNs with less than 10 nodes), being that their costs are prohibitive and do not allow a flexible adaptation of the signal processing algorithms at the sensor9 level. On the other hand, the systems presented in [87,88,89] aim at providing a fairer balance between deployment costs and achieved accuracy by relying on low-cost acoustic sensors that can only measure global aggregated levels of equivalent noise, but they enable a more pervasive installment of the WSN over the monitored area.

### 2.2. WSN for Land Monitoring

WSN technologies have found several application contexts in the field of soil monitoring. The ever increasing urbanization of the modern society is leading to a significant growth of the total amount of waste produced per year [90], which calls for adequate monitoring systems to supervise the storage, treatment, and recycling processes. Such systems should guarantee that some pivotal parameters (e.g., level of radiation, percentage of chemical and biological pollutants) are within the tolerated ranges and, whenever possible, prevent the illegal dumping of solid/liquid waste in the environment. As an enabling technology of the emerging Industry 4.0 paradigm, WSNs have been employed to monitor nuclear storage facilities [91,92], the disposal of chemical materials and waste [93], and, more generally, harsh industrial environments [94,95]. A common challenge faced in all these scenarios is represented by the extreme environmental conditions in which the WSNs are deployed. More specifically, very high temperatures, hazardous gases and the possible presence of chemical acids in the surrounding of sensors can severely impair the correct sensing capabilities of the WSN. Building a reliable and robust WSN monitoring system in harsh environments has been a topic of interest in the literature [96]. The proposed solutions typically augment the sensing systems by adding multiple backup sensors equipped with supplementary modules. This, however, comes at the price of an increased power consumption, which in turn calls for the necessity of proper energy harvesting technologies [97,98].

WSN monitoring systems play a crucial role also in fostering the prevention, early detection and quick management of natural disasters. Critical though recurrent phenomena such as wildfires can be combated by using WSN monitoring systems based on long-range (LoRa) wireless communication technology, which provides sufficient coverage for small and mid-size areas [99,100,101]. WSN monitoring systems turn out to be effective also for landslides prediction [102]. Due to the several factors that influence these phenomena (e.g., characteristics of soil, altitude, vegetation, etc.), the data collected by the WSN should be fused with geotechnical and hydrological models [103] and further processed within GIS in order to perform accurate analyses [104].

Precision agriculture is another important field of application for WSN-based monitoring systems. Introducing sensing technologies into the basic farming processes, from the optimal provision of soil nutrients up to an efficient management of the irrigation systems and fertilizers, is pushing agriculture toward the new emerging dimension of smart agriculture (also called Agriculture 4.0) [105]. A lot of work has been devoted to the adoption of WSN technologies to monitor the main soil parameters such as the moisture, temperature, pH, and wind direction [39,106], as well as the quality of crops [107] and the efficiency of the irrigation systems [108,109]. From the surveyed literature, it appears that the Arduino platform is one of the most adopted technologies to integrate a variety of soil sensors, while Tiny OS is the leading operating system installed on the WSN nodes. Among the main open challenges, it is worth mentioning the trade-off between the optimal deployment of sensor nodes to guarantee full coverage of the farming area and the choice of the low-power wireless communication technology to overcome attenuations and blockages due to the presence of dense crops.

### 2.3. WSN for Marine and Water Monitoring

During the last decades, marine environments have been severely threatened by effects related to anthropological activities such as tourism, urbanization, and industry [35,110]. Compared to traditional systems based on oceanographic research vessels, WSNs have provided dramatic improvements in terms of real-time analyses and monitoring of marine and coastal areas: on the one hand, the high cost associated with the startup and maintenance of the vessels is completely avoided [111]. In addition, the higher sensing resolution in both time and space promotes a more timely response against unexpected critical events such as flooding or water contamination [112]. A typical WSN-based marine monitoring system consists of a set of nodes deployed near the coast and/or in strategic points of the sea surface. The general architecture of each WSN node comprises a floating support (usually a buoy) to isolate the main electronic parts and RF communication modules from the water along with a sensing subsystem equipped with underwater sensors measuring different physical parameters such as pH, temperature, pressure, level of salinity, turbidity, oxygen density, and chlorophyll. Differently from land or air applications, WSNs operating in marine environments face three main additional challenges: (i) sensor nodes need to be protected against corrosion and adverse conditions such as tides, high waves, cold/hot currents, and typhoons, which undermine both the stability of nodes and sensing accuracy; (ii) preserving and harvesting energy is of utmost importance since sensor nodes are often deployed in unapproachable points of the sea and use long-range power-hungry wireless communication protocols to send data to the monitoring centers; (iii) underwater communications are highly unreliable due to extremely low channel capacities and high signal attenuations experienced when communicating through water. To address the first issue, biofouling protection capabilities have been added to the underwater sensors [113,114], and more advanced flotating buoys have been designed to better support the sensing nodes [115]. The energetic problems have been tackled by using both overwater [116,117] and underwater solar energy harvesting technologies [118] as well as by leveraging the kinematic energy from waves [119]. For underwater communications, acoustic waves represent the mostly adopted technology, even if they still suffer from high attenuation, significant delays in propagation, and fading effects. In some cases, a combination of optical, acoustic, and electromagnetic communications should be used to overcome the unreliability of the underwater links [120,121], possibly coupled with advanced routing [122] and hop-counting strategies [123] for improved efficiency.

Monitoring the quality of fresh-water courses and related drinkable water supply systems is another appealing application of WSN technologies. Providing clean and controlled drinking water typically involves a manual collection of water samples followed by intensive laboratory-based analyses. These approaches are, however, highly inefficient and expensive and cannot provide real-time information about water quality, preventing the possibility of timely identifying accidental or malicious contamination. In this respect, WSNs provide an important shift in the monitoring paradigm, enabling a real-time, low-cost analysis of the main water parameters (e.g., turbidity, pH, conductivity, etc.) with improved spatial and temporal resolution [36,124]. Given the absence of accurate biological and chemical sensors on common low-cost WSN nodes [125,126], most work in the literature has focused on the design of advanced contamination detection algorithms that properly fuse the different data collected from multiple sensors [127]. This kind of approach intrinsically suffers from increased false alarm rates, which in turn calls for more sophisticated methods that correlate the decisions with additional information coming from other external sources [128].

### 2.4. Main Challenges and Limitations of WSN Environmental Monitoring

Despite their transversal applicability to almost all the main environmental contexts (from air to land and up to sea), WSN technologies are still subject to a number of important issues that should be carefully considered when employing them for monitoring purposes. The main open challenges and limitations include:

- *Power Management and Node Lifetime:* The limited autonomy of WSN nodes, equipped with reduced-capacity batteries, is a major concern for WSN-based environmental monitoring systems, especially when nodes are deployed strategically though hardly accessible areas. Sophisticated strategies need to be conceived to ensure minimum energy consumption, with a particular focus on the most demanding RF components. Two main approaches are typically followed: (i) developing energy-efficient algorithms and communication protocols; (ii) using energy-harvesting techniques to restore energy based on solar cells, piezoelectric vibration-based devices, etc. Recently, new approaches for wireless energy replenishment started to be explored, relying on the availability of an additional set of mobile rechargeable units to prolong the lifetime of WSN nodes [129,130]. Preliminary results showed that such methods can significantly extend the duration of the sensing campaigns, thus representing a promising solution for WSN-based environmental monitoring [131].
- *Communication Range:* Communications in WSNs are typically performed using relatively low-power wireless technologies (e.g., ZigBee), which can only guarantee limited coverage. In most environmental monitoring scenarios, the harsh propagation conditions could lead to frequent obstructions or blockages of communication signals, potentially jeopardizing the whole sensing process. Some attempts have been made to improve the connectivity by studying the optimal placement of sensors under the assumption of some underlying wireless channel model. However, the practical solution adopted in most real deployments is still to increase the density of nodes in the WSN, with a consequent increase in the overall cost. In recent years, the use of connected dominating sets started to emerge as an effective way to reduce routing costs between sensing nodes and to generally improve the communication range, especially when WSN nodes are unevenly distributed over the target area [132,133]. Such approaches can be thus used to support node deployment and to make data collection/dissemination within the network much more efficient [134].
- *Sensor Data Quality:* Typical low-cost physical and chemical sensors employed for environmental monitoring return measurements that can be highly inaccurate, especially in the presence of miscalibration of the sensing units. Assessing the quality of the collected data becomes a priority when multiple heterogeneous sensors are used to monitor the same environmental phenomenon. Advanced outlier detection and data fusion algorithms are currently under investigation in the literature to avoid instances of a few unreliable measurements compromising the entire acquisition campaign. Accurate time synchronization of all the collected data represents another crucial aspect for obtaining reliable analyses [135]. As a prerequisite for most data-fusion algorithms [136], temporal information is combined with positional information to spatially contextualize the sensed data and outline the spatio-temporal correlations existing among them. This is of particular interest when dense WSNs are employed, for instance, to monitor environmental phenomena over very small areas [137]. In these cases, measurements collected by each WSN node are likely correlated among each other as well as with the measurements carried by neighboring nodes in the network. Notably, accurate clock/data synchronization is of utmost importance when some relevant environmental parameters, inferred from data, are used to detect possible violations of safety-critical thresholds in real-time or used to feed numerical prediction models to assess the possible evolution of phenomena both on a temporal and geographical scale [138,139].
- *Reliability and Fault Tolerance:* Robustness against possible hardware, software, and communication failures is a crucial aspect for WSNs to be effective in environmental monitoring. Given the low-cost nature of sensor nodes, even common phenomena such as rain, humidity, and wind can induce circuitry faults or frequent system reboots. Guaranteeing a highly reliable WSN is of utmost importance, especially when monitoring dangerous environmental phenomena (e.g., wildfires, water contamination, radiation) in real time, which requires that any potential emergency be promptly reported to the competent authorities. Enhanced reliability and fault tolerance are typically achieved by introducing redundancy of the main hardware components and by designing proper routing mechanisms and topology control schemes.
- *Scalability and Cost:* Most of the main environmental phenomena usually occur on a large spatial and temporal scale, following highly dynamic evolution processes. Monitoring them would thus require scaling up the WSN so as to cover vast areas with a significant number of sensors. Unfortunately, it is not often possible to deploy a dense WSN over a large-scale environment, for both physical and economic reasons. This is widely confirmed by the reviewed literature, where it emerges that WSNs are mainly used for monitoring relatively small areas.

## 3. Environmental Monitoring Based on Unmanned Aerial Vehicle Technologies

**Figure 3:**
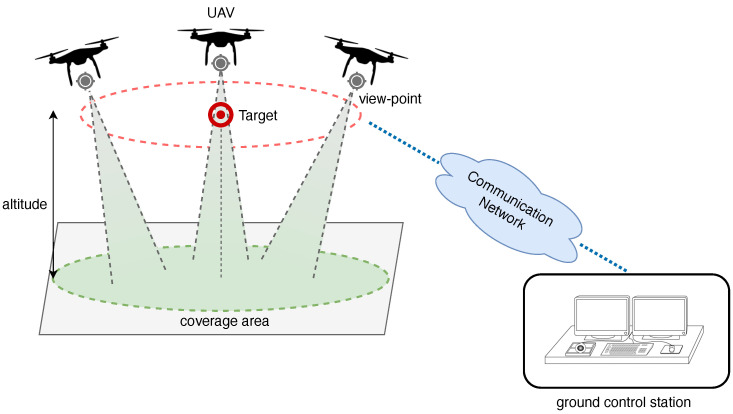
General architecture of an environmental monitoring system based on UAVs.

Over the last decade, UAV systems have progressively evolved toward a level of maturity that makes them powerful and versatile platforms for improving environmental monitoring tasks [40,41]. Figure 3 illustrates a general UAV-based monitoring architecture, which comprises a swarm of UAVs acquiring data over a specific monitored site defined by a set of target points, coordinated by one or more ground control stations, which are also responsible for the processing and preliminary analysis of the collected data. UAVs are available in the market under different models based on the type of propulsion (fixed wing or multi-rotor) and the maximum payload that can be carried. In Table 3, we report the main type of UAV platforms used for environmental monitoring purposes, together with their average coverage and main characteristics.

UAV nodes are equipped with a local microprocessor, an internal memory, a sensing and communication unit, as well as by some additional subsystems:

- A *navigation and guidance unit* responsible for obtaining real-time geolocation information using a GNSS receiver, usually coupled with a set of inertial and odometry sensors (e.g., accelerometer, gyroscope, etc.), as well as ensuring that a predefined trajectory is followed according to a specific path-planning strategy (mission).
- A *propulsion unit* using engines, motors, and batteries as power sources, as well as propellers or propulsive nozzles to generate and control the UAV motion.

UAV systems exhibit excellent monitoring and sensing capabilities: indeed, thanks to their aerial inspection ability and flexible characteristics (small size, rapid maneuvering), wider areas of interest can be covered in a timely manner, guaranteeing at the same time accessibility also to sites that would be inaccessible for other technologies such as WSNs or fixed monitoring stations. Although optical and multispectral/hyperspectral cameras remain the primary source of data on UAV nodes, other sensors such as thermal cameras, LIDARs, and gases sensors can be mounted as well. We summarize in Table 4 the most common types of sensors used in UAV-based environmental monitoring systems and refer the interested reader to [140] for a comprehensive review. It is worth noting that some of the sensors can be equipped only on specific types of UAVs, mainly due to the still too-high weight, cost, and maintenance of some components.

Compared to the more traditional manned airborne systems or satellite systems, UAV systems represent a cost-effective technology able to provide acquisition campaigns at an increased spatial and temporal resolution, offering the possibility to accurately track the dynamics of the main environmental processes occurring at very fine scales. High flexibility and easy adaptability to different application contexts make them suitable candidates to meet some of the crucial requirements of environmental monitoring: first, they enable real-time inspection of targeted areas thanks to their ability to perform rapid and repeated acquisitions of environmental data. Second, monitoring of hazardous or contaminated sites becomes possible using UAVs, with practically no risks for human operators. Third, the possibility to quickly reschedule UAV missions in the presence of unfavorable meteorological conditions (e.g., cloudy, rainy, etc.) allows for overcoming the main limitations of both airborne and satellite systems. In addition, UAV operations can be in principle guaranteed over the whole day and not limited only to specific hours as is the case of most satellite systems, enabling in turn a continuous environmental monitoring. Particularly, the costs involved in the deployment of a UAV-based monitoring system are not a limiting factor as for airborne or satellite systems: indeed, the main expenses are only linked to the initial investment in terms of hardware, software, and on-site equipment.

The above-discussed advantages, combined with the continuous evolution in the miniaturization of electronic and sensor technologies, led UAV systems to be widely applied across different domains of the environmental monitoring. In the following, we provide a review of some representative approaches proposed in the literature, classified based on their fields of application: (i) air monitoring, (ii) land monitoring, and (iii) water/marine monitoring. On the basis of the reviewed literature, we then conclude the section by highlighting the common challenges and the main limitations of UAV-based monitoring systems.

### 3.1. UAV for Air Monitoring

Although UAV technologies are mainly recognized for bringing disruptive enhancements to land and maritime monitoring, their aerial inspection capabilities have opened a new frontier also in the field of air pollution monitoring. Some experimental campaigns, conducted in crowded urban areas, revealed that the level of expansion of the main atmospheric aerosols and gases varies dramatically with the relative elevation from the emitting source. Therefore, monitoring the air pollution only at a ground level (in the order of 1–5 m) could not be sufficient to carry out an accurate air quality assessment. In these contexts, one or more UAV nodes equipped with dedicated gases sensors (as those described in Table 2) can be employed to monitor and track the pollutant concentrations at different altitudes [141,142], delivering at the same time richer real-time information that can be stored and used for long-term analyses [46,143]. Given the quite limited payload that needs to be shared among multiple sensors, some research efforts have been devoted to the design of lightweight gases sensing units for UAV nodes [144,145]. Finding the optimal placement of air monitoring sensors on UAV platforms is another challenging issue, given that the sampling and estimation processes are strongly affected by the dynamic nature of the wind and by the vortex fields generated by the propellers [146,147]. The high spatial and temporal sensing capabilities of UAV nodes have been also employed to build fine-grained air quality index (AQI) maps. Accurate profiling of the air pollution in urban/suburban environments can be achieved in nearly real time [148,149,150], especially when the AQI maps are combined with statistical plume models (such as the Gaussian) that characterize the physical dispersion in the air [151].

UAV nodes equipped with miniaturized infrared thermal cameras offer the possibility to enhance tasks related to the microthermal environmental monitoring, which consists in providing high-resolution analyses of the land surface temperatures and their variations, even at a microscopic level [152]. This information, combined with data related to humidity, solar radiation, and wind speed, is used to support important services such as weather forecasting or to infer the chemical composition of clouds (e.g., near a volcano, or after a chemical disaster) [153].

In recent years, some studies started to investigate the potential of exploiting the flexibility of UAV sensors to perform spatial, temporal, or spatio-temporal spectrum sensing, with the aim of revealing and possibly localizing sources responsible for electromagnetic pollution [154]. The same principle has been applied for monitoring acoustic noise in urban and suburban areas, leveraging UAV nodes equipped with an array of microphones. However, the proposed solutions are still at a preliminary stage, with such contexts being much more challenging to handle due to the presence of additional disturbances such as the wideband noise induced by the propellers and the narrowband noise generated by the engines [155].

### 3.2. UAV for Land Monitoring

UAV technologies have radically revolutionized the panorama of land environmental monitoring, enabling new horizons that were unconceivable until only a decade ago. The undisputed widespread use of such technologies across a wide range of soil monitoring tasks is strictly correlated with the progresses in the miniaturization and portability of RGB cameras and multispectral/hyperspectral imaging technologies, which allow to bridge the gaps with conventional satellite or airborne remote sensing platforms while providing a cost-effective way to obtain data at high spatial and temporal resolution [156,157]. By inspecting target areas at a very fine scale, UAV platforms are foreseen as potential tools to detect and counteract the illegal dumping of solid/liquid waste in the environment [158,159], for actively monitoring the operations in existing landfills [160], and for general prevention of soil contamination [161]. Preventing the diffusion of unauthorized constructions or their illegal demolition is another important application field for UAV-based monitoring systems [162]. From the surveyed literature, it emerges that these monitoring problems can be addressed using statistical change detection approaches operating on a temporal series of images or video sequences. Specifically, the availability of accurate a priori geometrical information (e.g., digital surface maps), possibly in combination with real-time kinematic information from the onboard inertial sensors, is a necessary ingredient for effective detection of soil contaminants with reduced false-alarm rates. Furthermore, since high resolution UAV images could likely trigger many undesired changes, advanced registration algorithms that properly align images acquired at different elevations and under different perspectives should be used at the pre-processing stage. In particular, when processing spectral data, change detection algorithms should expect higher in-class variances due to different acquisition conditions (e.g., illumination, shadows) and more complex scenes at hand [163].

UAV systems represent an indispensable technology to support onsite real-time monitoring of wildland areas subject to risks of natural hazards or disasters. Thanks to their aerial capabilities, environmental operations can be quickly conducted even in situations in which ground-level technologies could not be applicable and human intervention is too dangerous. The most prominent use case of UAV-aided natural disaster monitoring is the recurrent problem of forest wildfires [44]. Typically, high-resolution images acquired by RGB or infrared cameras are combined with information from the onboard inertial sensors and processed through advanced image processing algorithms to detect wildfires at their early stages and track their temporal evolution. Traditional computer vision approaches such as median/Gaussian filtering, image segmentation, and color analysis (in both RGB or HSV spaces) have been successfully applied to perform smoke detection and successive identification of the fire location in terms of altitude, latitude, and longitude [164,165]. When the ground control stations are equipped with significant computational power, deep learning algorithms such as convolutional neural networks and deep neural networks with an underling YOLOv3 architecture can be used to achieve improved flame and smoke detection performance at reduced false-alarm rates, even in the presence of adverse cloud and sunlight conditions, as well as undesired reflections from objects in the scene [166,167,168]. Image/video-based analytics have proven their effectiveness mainly for wildfires localized in relatively small areas. When the wildfire spreads over much larger scales, different measurements (e.g., temperature, wind) collected by a swarm of UAVs are fused within advanced filtering approaches (e.g., Kalman-based) that include wildfire propagation models such as the Rothermel or the Canadian forest fire behavior [169,170]. In addition to data processing, suitable distributed control frameworks need to be devised to design time-varying trajectories that enable a close monitoring of wildfires through multiple coordinated UAVs while minimizing the risk of in-flight collisions or damages, as well as to reduce the total number of transmissions toward the ground control stations [171,172]. Based on these advanced control schemes, some proactive approaches have started to appear in the literature that exploit the payload of UAVs to drop fire retardants or extinguishing agents at the epicenter of the wildfire [173].

Besides wildfire monitoring, UAVs have been employed to counteract geological hazards such as landslides using both optical [174] and thermal remote sensing techniques [45], and even to perform vulnerability analyses after natural disasters such as tornados [175]. Apart from the benefits brought to each individual application context, a common advantage of using UAVs in natural disasters monitoring, from prevention to recovery, is their ability to rapidly reproduce high-resolution maps of the target areas, a task usually called land-use land-cover mapping (LULC). Such maps, which can be either two-dimensional (surfaces) or three-dimensional (volumes), are at the basis of any emergency response application supported by a UAV system [20].

Proliferation of UAV systems brought new opportunities also in the field of vegetation analysis for both natural and agricultural environmental aspects [176]. Assessing vegetation health is a complex process that requires a combination of several indexes extracted from multiple sensors (ranging from optical images, infrared, and multispectral/hyperspectral). UAV platforms have been used to retrieve important information from natural habitats and ecosystems, including the monitoring of plant infection [177], average tree mortality [178], and level of diffusion of serious diseases such as the Xylella fastidiosa [179], with a granularity that can even reach the tree-level. Across all the considered technologies, hyperspectral imaging turned out to be a preferable tool to rapidly detect the level of vegetation stress based on the examination of pigments and chlorophyll [42]. On the other hand, a combination of data from optical cameras and LIDAR represents the most reliable solution when a 3D reconstruction of trees and crops is the main objective of the monitoring task.

Precision agriculture is another application context that can reap great benefits from the use of UAV-based monitoring. Compared to more traditional systems (e.g., satellite-based), UAVs provide field-level analyses that can be fruitfully exploited for the early diagnosis of agricultural problems, enabling in turn timely corrective actions from the farmers and, consequently, a significant reduction in both costs and environmental impact [180]. Prominent examples concern the use of UAV platforms to monitor the status of crops [176] and the quality of the soil [181], which allows for obtaining accurate predictions of the ultimate yield. From a technological point of view, RGB and thermal data have proven their usefulness for quantifying the main soil moisture contents, while accurate estimation of the water contents in the subsurfaces can be achieved by additionally exploiting mathematical models (e.g., the Soil Moisture Analytical Relationship) that link measurements collected at the surface to the parameters of interest [182].

### 3.3. UAV for Marine and Water Monitoring

Coastal and marine environments represent exciting application fields for the use of UAV monitoring technologies. Despite that most of the aerial remote sensing techniques in these contexts are based on satellite or airborne systems, mainly due to their wide coverage, UAVs open up a new set of significant opportunities to overcome the still too-limited imagery resolution, as well as the coarse and often discontinuous acquisition rate. By exploiting the improved spatial and temporal resolution and the availability of multiple sensors, UAVs can be used to carry out in-depth water quality analyses based on multiple correlated parameters [183]. More specifically, peculiar characteristics of the water surface reflectance captured by hyperspectral cameras can be used to detect suspended solids [184], particulate matter [185], and even toxic agents (chemical, biological) [186], while machine learning tools (e.g., SVM) turned out to be very powerful solutions to identify the presence of oil spills in optical images [187,188]. Using the Nemerow index and traditional regression techniques, the presence of smelly water can be readily identified [189], and the level of water transparency can be assessed in near real-time [190]. Spectral cameras carried by low-cost UAVs have been also used to monitor the sedimentation levels in natural reservoirs, and to assess the presence of submerged vegetation and algae species that are considered microbiological indicators of good water quality [191]. Notably, UAV technologies recently started to be adopted as a means to counteract the main phenomena of seaside degradation such as the dispersion of litters on the beach [192], or the uncontrolled dumping of plastic debris that are seriously threatening the aquatic wildlife [193].

The integration of water observations collected from UAV sensors with hydrological models allows for significantly enhancing the accuracy in describing the dynamics of rivers, lakes, and seas, enabling in turn a more effective monitoring of critical phenomena such as inundations and floodings. A number of proofs of concept have been proposed in the literature to demonstrate the feasibility of applying UAV optical techniques to perform distributed estimation of kinematic parameters such as the velocity of surface flow fields (e.g., using Large-Scale Particle Image Velocimetry) [194], which are used to delineate candidate flooding zones. Interestingly, some experiments revealed that an accurate survey can be obtained by just letting the UAVs hover for a few seconds around the target area, provided that orthorectification and photometric calibration phases have been correctly performed. When advanced deep learning algorithms are used to process and classify RGB images, accurate mapping and tracking of the flood routing and its probable extent can be inferred in near real-time [195]. Proper fusion of data coming from different sensors and an accurate modeling of the main hydrological parameters and their mutual dependencies remain the main open challenges to be faced before UAV platforms can be fully considered to support civil protection agencies in critical water monitoring tasks.

### 3.4. Main Challenges and Limitations of UAV Environmental Monitoring

The huge potential of UAV technologies is largely demonstrated by the several environmental application fields in which they brought not only significant improvements, but also radical revolutions. At the same time, we are witnessing the emergence of a significant number of methodologies based on specific combinations of hardware technologies (e.g., sensors, platforms) and algorithms (either for path planning, sensor calibration, or data processing) that are tailored only to the peculiar needs of each selected case study. In this respect, a major substantial challenge that should be addressed concerns the proper harmonization and standardization of processes involving the application of UAVs for environmental monitoring purposes. In addition to such a general challenge, from the analysis of the reviewed literature, some common though important open issues tend to emerge:

- *Policy and Regulations for UAV Operations*: The operations of UAV platforms are subject to regulations and restrictions imposed by governments that generally differ across different countries. Such limitations are imposed to guarantee the general public safety (especially in the presence of damages of the UAV platforms) and to ensure that the UAVs do not interfere with other aerial systems that share the same flight areas. To date, most of the regulatory frameworks do not allow fully autonomous UAV missions but require the presence of a licensed pilot to carry out even the most basic operations. Since these requirements inherently restrict the minimum distance at which UAV platforms can sense environmental data (known as Ground Sample Distance (GSD)), they represent one of the greatest obstacles toward a diffuse use of UAVs for environmental purposes.
- *Sensor Calibration and Error Correction*: Most of the lightweight sensors designed for UAV platforms typically experience significant geometric and spectro/radiometric limitations, calling for the need of adequate self-calibration and pre-processing procedures. Radiometric calibration includes several steps (such as the adjustment of colors, removal of noise, and deblurring) and requires the presence of spectral targets with known reflectance properties. Unfortunately, such a process is severely threatened when UAVs operate in adverse weather conditions (rain, wind) due to induced undesired spectral effects such as variable illumination, alterated reflectivity of materials, partial absorption, etc. On the other hand, the rapid maneuvers and frequent changes in flying altitude and orientation typical of the motion of UAVs introduce undesired impairments such as lens distortion and misalignment of the fundamental camera parameters (e.g., focal length, distortion coefficients, etc.) that should be compensated by means of a geometric calibration process. The overall correction process is known as *orthorectification* and represents one of the main research topics [196].
- *Flight Time and Path Planning*: The limited flight time of UAV platforms represents another crucial aspect that should be carefully taken into account when planning an environmental sensing campaign. This problem can be generally managed in two alternative ways: one possibility is to devise optimized path-planning strategies that take as input the extent of the area under investigation and the energy constraints of each involved UAV node and produce a set of trajectories (expressed as sequences of points of interest, as shown in Figure 3) that try to guarantee a satisfactory trade-off between coverage, sensing accuracy, and total duration of the data acquisition campaign. In this respect, recent studies have demonstrated that even the specific geometry of the flight path, passing through all the selected points of interest, can also have a strong impact on the achievable coverage and timely data acquisition capabilities of UAVs [197]. In particular, simple geometric flight patterns easily meet short path length and minimum mission execution time requirements but may conflict with other requirements such as energy consumption, being that short and simple paths are more likely to contain abrupt maneuvers, which in turn consume more energy [198]. A second possibility consists in leveraging the recent advances in lightweight battery technologies, which promise extended flight durations from about 1 h up to 5 h if solar-panel-based energy supplying systems are also integrated onboard. Overall, the experimental campaigns conducted so far have revealed that current UAV technologies can be considered cost-effective monitoring tools mainly for areas of quite limited extent (0.2 km${}^{2}$), while for larger areas, other technologies need to be adopted as complementary solutions.
- *Localization and Tracking*: Accurate estimation and tracking of the position and orientation information of UAVs over time is a fundamental prerequisite for all tasks involved in the monitoring process, from the initial pre-flight path planning until the data processing and subsequent analyses stages. On the one hand, ground control stations need to accurately predict UAV trajectories in order to design distributed control strategies that effectively coordinate the monitoring operations, especially in the presence of swarms of UAVs, without the risk of collisions or damages. On the other hand, any aerial photogrammetry-based method strongly depends on the accuracy of the *georeferencing* process. This task, also called registration, consists in associating the collected digital images to physical locations in the space through the definition of a set of ground control points (GCPs). Current practices in UAV enviromental monitoring consider the use of onboard GNSS and inertial measurements combined with the navigation and guidance unit to directly determine the UAV’s position and orientation [199]. However, such solutions turn out to be inaccurate or even unavailable in some practical operational scenarios since most of the hardly accessible sites monitored by UAVs are usually also GNSS-denied environments.

## 4. Environmental Monitoring Based on Crowdsensing Technologies

**Figure 4:**
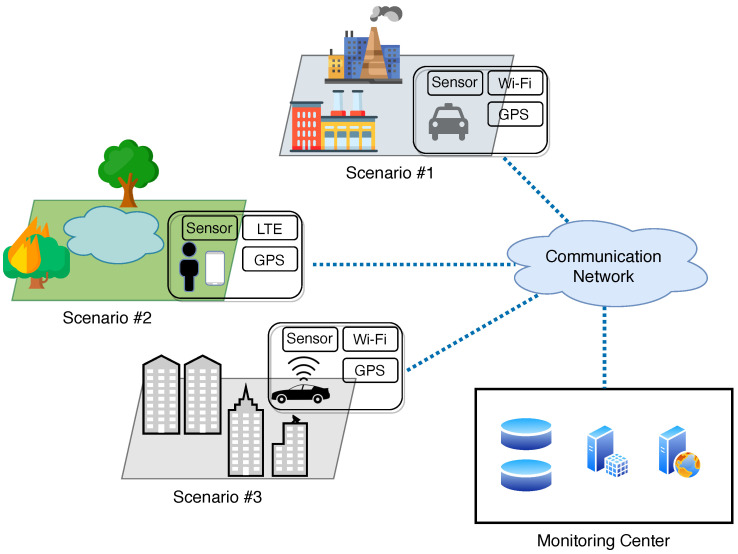
General architecture of an environmental monitoring system based on crowdsensing.

The pervasive, almost ubiquitous spread of smart mobile devices (smartphones, smartwatches, wearables, in-vehicle, etc.) featuring enhanced wireless communication capabilities and a rich set of built-in sensors (e.g., cameras, GPS, accelerometers, microphones) is progressively pushing the well-known benefits of the crowdsensing paradigm, in terms of large-scale sensing and information sharing, also into the context of environmental monitoring [47,50]. The general architecture of an environmental monitoring system relying on crowdsensing technologies consists of a pool of mobile smart devices leveraging their embedded sensors to collect different environmental data across different areas of the territory, according to the activities of the specific users (e.g., people moving in an urban center, vehicles traveling in forest or coastal areas, etc.), which are then sent to dedicated monitoring centers that are responsible for storing, integrating, and analyzing the huge volume of crowdsensed data, as shown in Figure 4. Sensed data are generally transmitted using different communication technologies, from ad hoc wireless networks (e.g., Bluetooth, Wi-Fi) to infrastructure-based networks (e.g., cellular 3G/4G/LTE). Thanks to the rapid evolution of portable sensor technologies, smart devices are equipped with an impressive number of built-in sensors that can be used to sense and monitor different physical parameters, e.g., electromagnetic fields, sound, temperature, humidity, etc. In Table 5, we summarize the most common types of sensors currently found in crowdsensing-based environmental monitoring systems.

Crowdsensing technologies offer enhanced monitoring capabilities compared to more traditional systems such as fixed stations and satellites. The key idea consists in building a *collective view* of the environment by exploiting data sensed by citizens in their daily routines. This can be practically implemented by using the concept of *crowdsourcing*: a formidable task such as the large-scale monitoring of the environment, traditionally performed by specialized and complex infrastructures, is distributed among ordinary users that leverage their own smart devices to sense data. In this respect, two different sensing modalities can be adopted: (i) participatory sensing in which users voluntarily collaborate to accomplish the sensing tasks, possibly receiving some kind of reward for their contribution; (ii) opportunistic sensing where conversely users do not need to undertake specific actions and are even unconscious of the sensing process that is passively carried out. The crowdsensing paradigm brings two main advantages to the environmental monitoring field. First, the frequent temporal and spatial variations of natural phenomena can be more accurately captured by fusing the *big environmental data* collected across separated spatial locations at different time instants. This huge amount of information can be used to extend the scale of the sensing campaign, overcoming the often limited spatial and/or temporal coverage provided by other existing monitoring systems without any additional deployment cost. Second, introducing human intelligence into the sensing process complements the information collected by the sensors with a much deeper understanding of the operational contexts [200].

Although the general architecture in Figure 4 may somewhat resemble that of a WSN-based monitoring system, profound differences can be found between the two technologies. First, WSN nodes are deployed over fixed locations and are tailored to specific types of environmental analyses. Their specificity usually leads to data of higher quality, but at the same time involves increased cost for the deployment and management of nodes. Conversely, since crowdsensing nodes are general-purpose mobile devices equipped with different kinds of sensors, they can be reused for different environmental monitoring tasks without requiring the deployment of specific infrastructures, thus representing an appealing cost-effective solution. Second, WSNs nodes are low-cost devices with very limited processing, memory, and energy, which constrains their local capabilities and makes it difficult to perform continuous monitoring tasks. On the other hand, crowdsensing nodes benefit from improved processing capabilities and the possibility of recharging their own batteries, which significantly extends their operational range. Another important difference concerns the limited monitoring scale of WSNs compared to crowdsensing. A study conducted in [201] revealed that about 100,000 WSN nodes would be required to enable environmental monitoring of a mid-size city, while guaranteeing full spatial coverage and sufficient connectivity with the monitoring stations. In particular, the inherent mobility of crowdsensing nodes (along planned or random trajectories) can be exploited to sample natural phenomena at an increased spatiotemporal resolution [202].

In the following, we provide a review of some representative approaches proposed in the literature, classified based on their fields of application (air, land, or sea). On the basis of the reviewed literature, we then conclude the section by highlighting the common challenges and the main limitations of crowdsensing-based monitoring systems.

### 4.1. Crowdsensing for Air Monitoring

Crowded urban areas are recognized among the main sources of worldwide air pollution, but at the same time are the perfect places where several crowdsensing nodes can be recruited [203]. People equipped with smart devices, vehicles routinely traveling along city streets, taxis, buses, and any transportation system at large are only some prominent examples of the multitude of crowdsensing nodes that can be exploited to estimate sources of air pollution and to infer their potential impacts on human exposure, allowing in turn to mitigate and prevent their negative side effects [204,205]. Thanks to the availability of a huge amount of urban environmental data, a number of pilot research projects have been funded in recent years with the aim of assessing the feasibility of air monitoring via participatory crowdsensing. The HazeWatch application, developed as one of the first crowdsensing-based approaches, is nowadays actively used by the National Environment Agency of Singapore [206]. GasMobile, CommonSense, Third-Eye, AirSense, and 3M’Air are other examples of monitoring systems that demonstrate the possibility of building accurate air pollution maps using only off-the-shelf sensors available on citizens’ smart devices [207,208,209,210,211]. Focusing on vehicles and road transportation systems as crowdsensing platforms, the paradigm of *drive-by* sensing has been coined, and interesting experimental campaigns have been conducted in New York City to first quantify the sensing power of crowdsourced vehicle fleets [212,213] and then assess how the different mobility patterns (either predictable or completely random) impact the discrete-time sampling process [214,215]. Some theoretical work considered the adoption of a network of crowdsensing vehicles and mapped the problem of estimating the air pollution levels into a problem of spatial field reconstruction from samples randomly gathered in a multidimensional space [216]. Improved accuracy and efficiency can be obtained when the correlations among the sensed data are explicitly considered in the model and the unsensed regions are properly characterized [217]. By exploiting analytical models for the variations of the air pollutants concentrations, a cost-effective balance between performance in terms of joint sensing accuracy and communication costs using a vehicular sensor network can also be achieved [218]. In the presence of a sparse number of crowdsensing nodes, compressed sensing techniques can be employed as viable tools to reconstruct accurate air pollution maps using only a small selected set of samples [219,220].

In addition to air pollution monitoring, an increasing number of environmental applications harness microphones embedded in mobile crowdsensing nodes to measure the levels of ambient acoustic noise (e.g., generated by an intense urban traffic) and to infer fine-grained noise maps by fusing the aggregated information at both geographical and temporal levels [221]. Citizens’ mobile phones are the primary sources of noise measurements used across a number of important projects such as NoiseTube [222], NoiseMap [223], NoiseSpy [224], and 2Loud [225], just to mention a few. In most of the considered experimental campaigns, it has been shown that recording the sound pressure signals at frames of about 1 s (with 48 kHz sampling rate and quantization between 16 and 32 bits) is sufficient to enable an accurate prediction of people’s exposure [226] and to localize the main sources of acoustic noise [227]. Compressive sensing techniques turned out to be effective also in recovering noise maps when the number of crowdsensed nodes was very limited and the available samples were incomplete [228]. Noise features can be estimated at a significantly improved granularity (e.g., road level) when the measurements collected by crowdsensing nodes are coupled with advanced noise simulation models [229]. Once the accurate and large-scale noise maps have been reconstructed, advanced analytics can be applied to support proactive interventions aimed at abating noise annoyances [230].

A less investigated but still very promising application field concerns the use of crowdsensing nodes to actively monitor the levels of electromagnetic pollution [231,232]. Received signal strength (RSS) measurements opportunistically gathered from surrounding Wi-Fi access points have been used to build accurate maps of the electromagnetic environment, providing real-time information on the instantaneous power levels of each transmitting source [233,234]. A recurrent issue in this field is how to ensure the trustworthiness of the identified electromagnetic pollution sources while explicitly taking into account the intrinsic inefficiency of the sensors (e.g., antennas) used by crowdsensing nodes [235]. Some work tried to tackle this issue by formulating the spectrum sensing problem as a robust optimization problem, using a generalized modeling approach to take into account the possible presence of incomplete [236], abnormal, or untrustworthy data [237], also including possibly malicious users [238]. Another interesting solution considered the application of a maximum likelihood ratio test over a binary hypothesis, where the non-null hypothesis denoted the effective presence of a non-negligible source of electromagnetic pollution. To explicitly include possible sensor inefficiencies, an expectation-maximization (EM)-inspired approach has been applied to alternate estimation of the probability of successful source identification with the estimation of the sensor efficiency. To further increase the source identification accuracy, the maximum likelihood ratio test can be repeated multiple times as a sequential probability ratio test (SPRT) [239]. Recently, some preliminary work investigated the adoption of deep learning approaches to cope with the presence of very few crowdsensing nodes, especially when operating in harsh propagation environments [240].

### 4.2. Crowdsensing for Land Monitoring

The widespread availability of crowdsensing platforms gathering environmental data across different physical locations (from urban to rural areas) can offer an important support for monitoring the state and quality of soil parameters and to combat land degradation at large. A successful example in this field is the Danger Maps project developed in China, which is a crowdsensing-based monitoring system whose primary goal is to stimulate citizens in reporting the presence of sources of soil pollution such as illegal garbage dumps generated by toxic-waste treatment facilities, oil refineries, and power plants [241]. A single alert can be quickly triggered by simply reporting a textual description of the pollutant sources, possibly together with pictures captured via the embedded camera. Following the same line of Danger Maps, a general paradigm called *citizens as sensors* was recently introduced, which aims at actively involving citizens in the fight against land degradation [242], from monitoring the quality of the road surfaces [243] up to facing the rising threat of trash dumping [244].

The ever-increasing number of smart devices disseminated worldwide, combined with the almost ubiquitous availability of mobile communication networks, is opening new opportunities for the use of crowdsensing monitoring techniques to foster the prevention, early detection, localization, and management of large-scale natural disasters. The main advantages reside in the possibility to exploit real-time and geolocated information provided by users to delimit areas that deserve careful attention from the emergency response teams. For instance, some authorities started to consider the potential of such a paradigm to counteract wildfires [245]. While the idea of engaging citizens with their smart devices in the data-gathering process is relatively straightforward, several practical aspects need to be taken into account to guarantee timely and accurate detection of wildfires events, so as to minimize the probability of large-scale damages. More specifically, the crowdsensed data need to be properly processed to extract meaningful information: first, a preliminary coordination strategy needs to be conceived in order to identify the most appropriate type of data to be collected and, consequently, the characteristics of the candidate crowdsensing nodes. Then, the acquisition campaign should be carried out by including appropriate mechanisms to manage underlying non-idealities such as sensor failures and user errors, both accidental and intentional [246]. At the end, some kind of pre-evaluation of the collected data is required to select the most informative sources: Naive Bayes classification has been used to rank the reported data based on the user credibility [247], while multiple binary hypotheses tests have been employed to estimate the probability that the same wildfire event occurred on multiple locations of interest [248]. Social networks represent another valuable source of crowdsensed data but require additional data mining techniques to convert users’ public posts (e.g., Facebook, Twitter, Instagram, …) into meaningful features that can be used in the processing steps [51,249,250]. From an algorithmic perspective, the support vector machine (SVM) classifier has been largely used to map posts over social networks into textual features [251], possibly in combination with a natural language processing method such as the Bag-of-Words to extract additional information conveyed through shared videos [252]. Furthermore, some modeling approaches such as logistic regression may be required to track how the information flows across different groups of users and assess its correctness [253]. To generate knowledge from the aggregated data while satisfying the real-time requirements of wildfire management, efficient data-fusion techniques that operate over short time windows (comparable with the dynamics of the wildfire) must be applied to combine the different categories of reported data and simultaneously filter out any redundant information. The final analyses and visualization phases are responsible for superimposing the positions of identified wildfires on real-time maps and to infer the envelope of the interested areas, predicting the possible evolution of the fire based on propagation models fed with local meteorological data [254].

Besides wildfire monitoring, participatory crowdsensing has been employed for near real-time detection of other disaster events such as earthquakes [255], landslides [256], and floods [257]. A common approach in these contributions is to consider only sensor readings with an associate position information (either from GPS or cellular networks). Since such phenomena cannot be easily characterized with a single analytical model, stochastic inference tools such as the particle filter are used to reconstruct a spatial model from the collected observations, which is then used to estimate an approximate location of the hazard event.

Very recent studies have foreseen crowdsensing monitoring techniques as indispensable components in the emerging contexts of smart agriculture [49]. Thanks to the evolution in the worldwide economy, even in underdeveloped countries, an increasing proportion of farmers have at least one smart device and would be willing to use it for further increasing their income [258]. Compared to other application fields, in which citizens may not have a specific expertise, the experience accumulated by farmers during their professional career represents an extremely valuable source of information that can be deeply integrated and used as a boosting component in most farming processes. Although this paradigm, also known as *farmers as sensors*, is still in its infancy, very promising directions have already been investigated, such as the identification of possible crop pests and diseases via deep learning approaches applied to recorded photos and videos [259,260], prediction of possible agricultural disasters due to adverse meteorological conditions [261], accurate estimation of the cultivated lands by exploiting farmers’ mobility [262], assessment of the quality of fruits [263], as well as for planning the annual production in all its phases [264].

### 4.3. Crowdsensing for Marine and Water Monitoring

Crowdsensing technologies represent a valuable resource to enhance the study and monitoring of seas, lakes, and oceans, with the aim of forecasting possible catastrophic events, to support marine-related activities, and to prevent the environmental degradation of any marine or coastal area at large. Interesting research projects have been funded in recent years, especially in the south of Italy: the University of Cagliari, in collaboration with the Mediterranean Sea authorities, designed and validated a crowdsensing-based monitoring system whose main goal is the safeguarding of coasts against the negative effects caused by the raising phenomenon of mass tourism [265]. By integrating feedback sent by users in the monitoring processes, the system is able to accurately predict the actual occupancy rate of bathing establishments and to identify potential sources of littering. The SmartWave project developed by the University of Palermo (Sicily) is another example of a crowdsensing-based prototype that aims at monitoring the main phenomena characterizing the marine environment. To do so, the major research challenge was to design a data fusion module able to standardize and properly combine the huge amount of heterogeneous, possibly incomplete data collected by citizens [266]. A pilot study has been conducted in Greece to investigate the impact of the COVID-19 pandemic restrictions on tourism demand, and a prototype platform that exploits data crowdsensed by volunteer tourists has been developed to monitor a 16,000 km extended coastal zone [267].

Participatory crowdsensing can play an important role also in predicting and minimizing the impacts of some catastrophic marine events. By integrating crowdsensing monitoring with the use of social media, a more accurate estimation of fundamental geophysical models for the propagation of oil spills in the oceans’ surfaces can be obtained. A prominent example has been reported in [268], where images related to the disaster of the Deepwater Horizon in the Gulf of Mexico were collected from Flicker and used to support the forecast of the GNOME model parameters. Interestingly, the use of metadata information (location and time) associated with the crowdsensed images brought significant improvements in the estimation of the rate of oil spill diffusion and the coefficient of diffusion while also highlighting important correlations between currents and surface winds. Another relevant application field is the context of flood risk management. Due to the severe climate changes experienced in the last few years, flood occurrences increased considerably, and containing their effects requires up-to-date and accurate knowledge of some critical environmental state variables [269]. Some studies have demonstrated that smartphones can estimate important parameters such as water levels, with an accuracy that can even achieve the cm level when the images captured by cameras are fused with the builtin orientation sensors [269,270]. The main drawback resides in the still too-involved operations that are demanded of the volunteers in order to carry out meaningful measurements. Moreover, few maps representing an updated overview of the flood risk areas are available.

Monitoring the fresh water sources and related infrastructures is another potential application field of crowdsensing technologies. Preliminary work investigated the behavior of the involved citizens when solicited to participate in the sensing campaign [271]. A data analysis conducted using the theory of planned behavior demonstrated that some demotivational factors could severely impact the willingness of citizens in reporting their collected information. To overcome such a drawback, a gaming approach for urban water crowdsensing has been designed and validated in [272], where citizens are provided with a kind of entertainment by means of a gamified interaction with the environment. Although promising, such an application context is, however, less investigated in the available literature.

### 4.4. Main Challenges and Limitations of Crowdsensing Environmental Monitoring

Undoubtedly, crowdsensing technologies represent a very versatile solution to improve most of the processes involved in the environmental monitoring tasks, as they transversally impact all air, land and marine scenarios. Compared to the other existing technologies, they provide two fundamental benefits: (i) *cost-effectiveness*, as the sensing processes are entirely run on users’ mobile devices, which leverage their embedded sensors to sense or generate data, and hence they do not require the deployment of additional infrastructures, eliminating the need for purchasing new monitoring devices; (ii) *scalability*, since the number of crowdsensing nodes recruited for the monitoring tasks can grow significantly according to the willingness of citizens to contribute to the measurement campaigns, enabling in turn a potential large-scale analysis of the main environmental phenomena thanks to the improved spatial coverage. However, from the analysis of the surveyed literature, it can be observed that some important research challenges still need to be properly addressed:

- *Incentive Mechanisms*: To be effective, crowdsensing-based environmental monitoring must rely on a sufficient number of users participating in the sensing campaign. Although the timely topic of environmental protection may stimulate the general interest, people can be reluctant in providing some kind of “access” to their own smart devices, for either ethical or private concerns. In addition, for some specific monitoring tasks, the sensing process could require an intensive use of processing and communication resources, resulting in an inevitable consumption of energy for users’ devices [273]. Indeed, users may be asked to move to specific target locations and to perform certain actions in order to accomplish the sensing task, possibly deviating from their planned routine. Therefore, suitable incentive mechanisms need to be devised for compensating users’ contributions and promoting their participation in the monitoring tasks. Research approaches can be categorized in two main groups: monetary incentive mechanisms, in which users are paid with a monetary reward [274,275], and non-monetary mechanisms where instead users are rewarded with alternative incentives such as gaming, social entertainment, or virtual credits (e.g., coupons) [276,277]. In the former case, the monitoring system has the additional burden of implementing suitable automatic strategies to select the more convenient users, usually based on the distance from the task location.
- *Task Allocation and Workload Balancing*: The goodness of the environmental monitoring process also depends on the way the related sensing tasks are allocated to users. There are indeed several factors that should be jointly considered. First, users may have very different skills and expertise, which in turn produces a significant diversity in the quality of the crowdsensed data [278]. This is in trade-off with the limited budget typically available by the monitoring centers, whose main goal is to maximize the quality of data while minimizing the incentives delivered to users. Thus, obtaining high environmental data quality under budget constraints is a complex problem that requires advanced task allocation algorithms able to select proper users while explicitly taking into account crucial factors such as the position of users, their reliability, and the involved sensing cost [279]. In this respect, different approaches are currently under investigation: a first possibility is to adopt learning-driven approaches, where the crucial information required in the task allocation problem is directly provided by users at the recruitment stage. Another category of approaches considers the spatial and temporal correlations existing among different environmental tasks and allows users to share sensed data and infer information from other related tasks. Besides these aspects, it should be also considered that since each individual user has a limited processing and communication capacity (due to limited battery and hardware constraints), the number of maximum tasks that can be completed on a daily basis is typically quite limited. To avoid burdening the users with a too-high number of tasks, proper workload balancing methods must be designed to quantify the maximum tolerable overload for each user and decide accordingly the best tasks to be allocated.
- *Data Trustworthiness*: A still-open issue in crowdsensing-based environmental monitoring is how to prevent participating nodes from contributing to unreliable data and potentially jeopardizing the sensing campaign. Generally, two main possible scenarios are distinguished: in a first case, data unreliability is mainly due to faults and defects in the users devices, which unintentionally provide corrupted data. On the other hand, malicious users may contribute with fake sensing data (e.g., fake GPS readings, fake images, …) just to earn the associated rewards, affecting in turn the integrity of the data collected by the monitoring system [280]. Some attempts have been made to counteract the former scenarios, using sophisticated algorithms (e.g., compressive sensing) that aim at detecting and correcting false or missing information [281]. The latter scenarios are much more difficult to handle and require appropriate reputation models that correctly rank the level of trustworthiness of all the users involved in the crowdsensing process [282]. Few works have also tried to jointly deal with malicious participants and corrupted sensor data by combining different reputation and trustworthiness metrics [283].
- *User Privacy*: Another important factor that could lower the willingness of citizens to participate in the crowdsensing campaign is the risk of compromising their privacy. On the one hand, the monitoring platform needs to know the location of mobile smart devices so that sensing tasks can be allocated on a minimum distance basis. This potentially reveals the user movements and may disclose his/her common routines. On the other hand, crowdsensed data may contain sensitive information such as private pictures or personal health information. To deal with the first issue, location-preserving mechanisms that aim at masking user position are currently under investigation [284]. For sensitive data protection, advanced anonymization techniques that either remove, obfuscate, or encrypt part of the reported information seem to be a promising solution, ref. [285], though there are still several drawbacks to be fixed.
- *Mobile Node Localization*: The correct aggregation and fusion of the big environmental data collected by crowdsensing nodes strongly depends on the accuracy of their position information over time. From task allocation up to data visualization over maps, almost all the processing steps involved in the environmental monitoring process are based on the underlying assumption that users have a certain knowledge of their own position. In most cases, however, such an information is simplistically deduced from the onboard GNSS receivers, without considering that the latter should be frequently switched off to save energy and, moreover, are highly inaccurate or completely unavailable in many operational contexts (e.g., urban areas). To overcome such limitations, fully adaptive localization algorithms based on advanced signal processing techniques need to be conceived, which aim at providing ubiquitous though accurate positioning by combining all the sources of information onboard (e.g., GNSS, inertial sensors, visual sensors) with that available from cooperation with other crowdsensing nodes as well as with the surrounding infrastructures (e.g., cellular base stations, other existing systems) [286]. In this respect, the almost ubiquitous connectivity together with the advent of the emerging fifth generation (5G) and beyond (6G) cellular communications is offering promising opportunities to achieve seamless centimeter-level positioning in all the diverse contexts that are found in the environmental monitoring domain [287,288].

## 5. Signal Processing for Environmental Monitoring

The vast number of technologies reviewed in the previous sections, associated with an ever-increasing amount of heterogeneous environmental data collected from different sensors across different domains (from air to land, up to sea), calls for the need of theoretical methodologies that guide the process of maximizing the information extracted from the monitoring systems, while guaranteeing at the same time satisfactory levels of cost, complexity, and scalability. Clearly, substantial differences exist between the more controllable conditions experienced in WSNs scenarios, where sensors are static and located at known positions, and the more dynamic UAV and crowdsensing scenarios, where conversely sensing nodes move along time-varying trajectories, leading to a number of issues that need to be addressed. In this respect, *signal processing* theory provides elegant analytical tools to model some of the main issues that characterize the environmental monitoring tasks by representing them in proper spaces where specific signal methods can be applied [289]. This allows a better understanding of the considered phenomena as well as a proper planning of the sensing campaign [290]. In this section, we provide a review of some important applications of signal processing techniques to environmental sensing, showing their key role in enabling (i) enhanced real-time (short-term) and long-term analyses of the environment in all its aspects (air, land, sea) and (ii) an efficient deployment of the monitoring systems over larger scales. We propose a harmonization of the existing literature by classifying the surveyed methods according to three macro categories, each corresponding to a fundamental step performed in the environmental monitoring contexts.

### 5.1. Optimal Sensor Locations for Environmental Sensing

In a nutshell, a generic environmental monitoring task can be interpreted as the problem of inferring accurate information about a physical phenomenon (e.g., air pollution, temperature variation, acoustic noise, …) that can be observed only through a limited set of sensors. This creates an intrinsic link between the sensed phenomenon and the position of the sensors and suggests that the ultimate accuracy in retrieving the desired information depends on the spatial distribution of the sensors.

#### 5.1.1. Linear Inverse Problems

Mathematically speaking, this relationship can be formalized through the definition of an *inverse problem*, which in the continuous joint time-space domain can be expressed as the following linear relationship (as opposed to non-linear inverse problems, which are much harder to handle and thus are approximated to linear inverse problems for the sake of mathematical tractability in typical environmental monitoring applications):

(1) $$y\left(p,t\right)=\int_{0}^{t}A\left(p,t,\tau \right)x\left(\tau \right)d\tau$$

linking the measured physical field y(p,t) at position $p\in R^{d}$ (with either d=2 or d=3) and time *t* with the underlying physical phenomenon x(τ) through the spatio-temporal kernel A(p,t,τ). Since in practice the phenomenon can be sensed only at a limited number of points in space (corresponding to positions of sensors) and over finite time instants, a discretized version of (1) is usually considered

(2) y=Ax

where $y\in R^{N}$ contains the values measured by a set of *N* sensors placed at $p_{1},\ldots ,p_{N}$, respectively; $x\in R^{K}$ denotes the parameters of the physical phenomenon to be estimated; and $A\in R^{N\times K}$, sometimes called the *sensitivity matrix*, describes the linear relationship between the phenomenon and the measurements. It is worth noting that (2) can be extended to model more complex scenarios. For instance, if a sampling kernel is applied to the measurements, y can be expressed as a linear combination of the physical field and, accordingly, the sensitivity matrix can be factorized as A=ΨΘ with Θ the physical field and Ψ the sampling kernel. In general, A is an arbitrary complicated matrix whose entries are functions of some variables affecting the physical phenomenon at hand (e.g., for radioactive emissions, A could depend on the weather conditions and on the type of materials) and on the position of the source generating the physical field. When (2) is tailored to air pollution scenarios, A is typically generated using the Lagrangian Dispersion Models (LDM), which emulate the pollutant dispersion using random particles moving along arbitrary trajectories in space and time [291]. The entries of x depend instead on the specific inverse problem at hand. If the main goal of the sensors is to localize potential sources of pollution, a reasonable choice of x could be the pair intensity-position of each source [292]. If instead sensors are used to infer the whole physical field from the collected measurements, entries of x can be a lower-dimensional representation of the phenomenon [293]. We refer the interested reader to [294,295] for a good overview of linear inverse problems.

#### 5.1.2. Sensor Placement Problem Formulation and Possible Solutions

**Figure 5:**
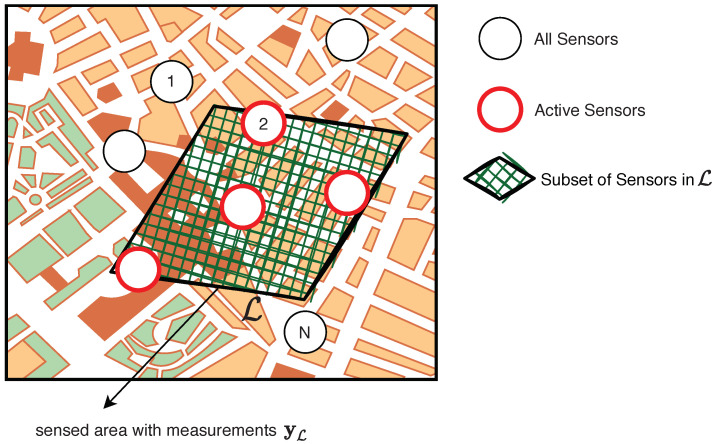
General setup of the sensor placement problem. White circles represent all the *N* sensors. Circles with red contour denote the active L≤N sensors producing the measurement vector $y_{L}$.

To formulate the general sensor placement problem, we consider the linear model in (2) and assume that a physical phenomenon x can be sensed with a number of sensors L≤N, as shown in Figure 5. The latter condition explicitly takes into account realistic contexts in which some of the environmental sensors may not be available, either due to damages or faults, or simply to preserve their energy, or for any other reason of temporary unavailability. Although this scenario may resemble a typical WSN-based monitoring system, in which sensor nodes can be carefully located during the deployment phase, it can be also adapted to UAV or crowdsensing scenarios when the mobile nodes can be assumed static for the limited duration of the sensing campaign. Let us denote with $L=\left\{\ell_{1},\ldots ,\ell_{L}\right\}$ the set of indexes corresponding to the actual measurements positions and $N=\left\{1,\ldots ,N\right\}$ the set of indexes associated to the overall sensors positions, with L⊆N. Accordingly, we can rewrite a reduced system of linear equations from (2) as

(3) $$y_{L}=A_{L}x$$

where $y_{L}\in R^{L}$ contains the reduced set of measurements collected by sensors in the set L, and $A_{L}\in R^{L\times K}$ is obtained by selecting only the rows of A corresponding to the indexes in L. The linear inverse problem can be then recast as finding an estimate of x using only L≥K measurements. We first notice that, given $y_{L}$, a solution $\hat{x}$ to (3) may be not unique, or may not even exist. For this reason, the estimation problem is usually formulated in a least-square sense as

(4) $$\hat{x}=\arg\min_{x}{∥A_{L}x-y_{L}∥}^{2}$$

with ∥·∥ denoting the norm operator. Assuming that $A_{L}$ is full-rank, a closed-form solution to (4) is given by

(5) $$\hat{x}=A_{L}^{†}y_{L}$$

with $A_{L}^{†}={\left(A_{L}^{T}A_{L}\right)}^{-1}A_{L}$ the Moore–Penrose pseudoinverse. Assuming that the measurements $y_{L}$ are corrupted by additive white Gaussian noise with zero mean and variance $\sigma^{2}$, we can express the mean squared error (MSE) on the estimation of x in closed-form as [296]

(6) $$\mathrm{MSE}\left(\hat{x}\right)={∥\hat{x}-x∥}^{2}=\sigma^{2}\sum_{k=1}^{K}\frac{1}{\lambda_{k}^{L}}$$

with $\lambda_{k}^{L}$, k=1,…,K denoting the eigenvalues of the matrix $T_{L}=A_{L}^{T}A_{L}$. From (6), it clearly emerges that the reconstruction of the environmental phenomenon x strongly depends on the spectrum of $T_{L}$, which is itself a function of the sensor locations indexed by L. Therefore, the optimal sensors locations can be obtained by solving the following constrained optimization problem:

(7) $$L_{\mathrm{opt}}=\arg\min_{L}\sum_{k=1}^{K}\frac{1}{\lambda_{k}^{L}}\;\;\;\mathrm{subject}\;\mathrm{to}\;\;\left|L\right|=L.$$

It is important to notice that the above optimization problem is combinatorial in nature, hence any brute force algorithm evaluating all the possible combinations would require an execution time that exponentially increases with the number of trial positions in L, rendering the sensor placement problem impractical even for moderate values of *N*. To overcome such a drawback, alternative approaches that trade off the optimality of the obtained solution for a reduced complexity have been investigated in the literature. Some preliminary work has applied approximate greedy algorithms with the aim of directly minimizing the MSE. However, it has been found that MSE does not lend itself to being easily optimized due to the presence of several local minima. This called for the need of finding surrogate cost functions that tightly approximate the MSE while being much more efficient to optimize [75,297].

The methodologies used to solve (7) can be classified in three distinct groups:

- Greedy algorithms;
- Convex optimization;
- Heuristic strategies.

Greedy algorithms are appealing due to their polynomial complexity and the possibility to infer a certain degree of confidence about the solution’s quality by exploiting the submodularity of selected families of objective functions [298,299]. On the other hand, no guarantee in terms of actual MSE can be provided, and the optimization processes typically involve the inversion of very large matrices [300]. Convex optimization algorithms transform the original problem into a convex problem by relaxing the boolean constraints in (7) into a convex set [301,302]. Unfortunately, there is no easy way to establish the tightness of the relaxation and, consequently, to ensure the quality of the suboptimal solution. Furthermore, when the involved sensors should be distributed over a quite large area (e.g., in crowdsensing-based monitoring scenarios), efficient computational methods such as the alternating direction method of multipliers (ADMM) or the accelerated proximal gradient method (APGM) must be employed to speed up the sensor location process [303]. Heuristic approaches significantly lower the computational complexity required by the exhaustive combinatorial search but lack any kind of guarantee compared to convex and greedy approaches, both in terms of the optimality of the solutions and convergence [304,305]. Besides the approximation strategies, another important aspect concerns the nature of the physical field to be estimated. If x can be assumed to be a spatio-temporally stationary stochastic process, as is the case for instance in flooding and long-term precipitation monitoring, more advanced approaches can be used to combine multiple snapshots using a proper deployment of fixed sensors that guarantee a cost-effective solution in terms of both processing time and energy [306,307]. On the other hand, if the physical field x is assumed to be non-stationary, a condition experienced in crowdsensing and UAV-based environmental monitoring tasks due to sensor mobility, higher-order statistics need to be computed from multiple snapshots and included in the optimization problem to obtain a proper dynamic deployment of the sensors, so that the dynamics of the physical field can be accurately tracked [308]. There exist also low-complexity approaches that solve the optimal sensor location problem under some specific conditions. This happens, for instance, when the monitoring system is inherently distributed and the sensors are sparsely disseminated over large target areas (a condition typically found in crowdsensing-based monitoring). For these scenarios, sparsity can be enforced in the optimization problem and exploited to obtain more convenient, relaxed versions of the original cost function, leading to the so-called sparsity-aware sensor location methods [309,310]. Given their promising performance, such approaches have stimulated rich research activity in the last years [311].

### 5.2. Sampling and Reconstruction of Environmental Phenomena

**Figure 6:**
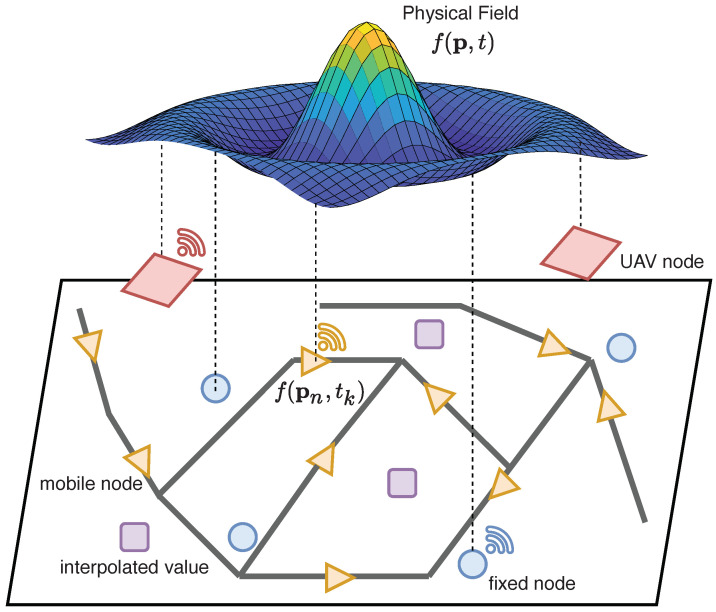
Sampling and reconstruction of an environmental phenomenon f(p,t). The WSN, UAV, and crowdsensing nodes measure the phenomenon at different physical locations. After reconstruction, the value of the field can be inferred also at unseen positions through interpolation.

One of the primary tasks performed by any environmental monitoring system is the accurate modeling of the specific natural phenomenon at hand, starting from a set of its samples. Prominent applications can be, for instance, the monitoring of water quality, for which suitable models able to predict the presence of pollutants need to be built. Typical samples may include the concentration of chemical pollutants or other related parameters such as the humidity, turbidity, or temperature. Other application fields are air quality monitoring in urban areas, analysis of the level of electromagnetic radiations, or the study of the soil moisture and related vegetation growth, just to name a few. In the signal processing literature, this process is known as spatio-temporal *sampling and reconstruction* of the specific physical phenomenon of interest. Thanks to the enhanced communication technologies available on sensor nodes, all the environmental data can be efficiently collected and further elaborated by centralized data processing algorithms, which leverage the link between the values of some selected environmental variables measured at specific locations and the position information itself to infer the behavior of the whole physical phenomenon, as shown in Figure 6. A physical phenomenon can be generally represented as a continuous function of two independent variables f(p,t), with *t* denoting the time and p the space variable (in either two or three dimensions). As discussed before, sensing nodes can only provide samples (or snapshots) of the continuous physical phenomenon collected at a finite number of locations ${\left\{p_{n}\right\}}_{n=1}^{N}$ and over a limited number of time instants $t_{k}=kT$, with k∈Z, and *T* being the chosen sampling period. Questions that naturally arise are whether the discrete samples $f(p_{n},t_{k})$ faithfully represent the physical phenomenon, and how the original function f(p,t) can be reconstructed from them, in either exact or approximate form. As an ubiquitous problem embracing several domains of the signal-processing literature, the process of reconstructing a physical field f(p,t) from a discrete set of its samples has received a lot of attention so far, and a number of different approaches have been investigated to solve it. The existing methodologies can be broadly classified in two main groups based on whether some a priori knowledge about the field f(p,t) is available or not. In the former case, more tailored approaches can be applied that benefit from the prior information (if e.g., f(p,t) admits a sparse representation or can be statically characterized according to a certain distribution) to achieve improved reconstruction performance. Conversely, in the latter case, the more classical tools from the sampling theory need to be adopted.

#### 5.2.1. Sampling and Reconstruction without Additional Information

The main results in terms of sampling and reconstruction root back to the celebrated Whittaker–Kotelnikov–Shannon (WKS) sampling theorem, which demonstrated that a physical field f(p,t) can be recovered with no errors if it is sampled *regularly* in the spatial domain with a sampling rate which is at least twice its bandwidth [312]. Clearly, the underlying assumption behind the theorem is that f(p,t) should have a limited bandwidth; unfortunately, this condition is hardly satisfied in practice, hence f(p,t) needs to be preliminarily filtered with a low-pass antialiasing filter. This operation intrinsically imposes that the field reconstructed from a limited set of filtered samples represents at most the optimal $L^{2}$ approximation of the original f(p,t). Such a pioneering work has been then extended to more specific scenarios or multidimensional domains [313]. A more exhaustive analysis of regular sampling over multidimensional fields (also called lattices) has been conducted in [314], where authors were able to provide a closed-form expression of the MSE. With the aim of keeping the sampling and reconstruction process as general as possible, interesting approaches based on a probabilistic quantization framework have been devised, which only impose minor restrictions on the possible variations of some selected environmental variables [315]. Almost along the same lines, the authors of [316,317] presented very flexible pdf-unaware estimation approaches to reconstruct environmental fields having a homogeneous spatial distribution, able to operate also in the presence of very limited bandwidths.

In practical environmental monitoring applications, guaranteeing a regular distribution of sensor nodes (either fixed or mobile) over a target area is seldom possible and, more often, irregular sampling strategies need to be adopted [318]. The most important result in the field of sampling theory for nonuniform (but deterministic) sampling has been provided by Landau [319], who was able to derive the necessary density of samples that should be used to guarantee an exact reconstruction of the environmental field. In [320], further studies have been conducted to take into account realistic conditions such as the presence of errors on sensor positions and signals with non-limited bandwidth. It has been shown that theoretical insights can be obtained only when restricting to specific classes of f(p,t) possessing the shift-invariance property, and when linear interpolators are used to reconstruct the whole field.

A deterministic deployment of the sensor nodes (either regular or nonuniform) may not be possible when the monitoring system operates in hazardous or remote areas (as is the case in most natural disasters scenarios). In such contexts, sensors are randomly scattered over target areas (for instance, through airdrop) and their sampling process is inherently stochastic. If samples’ positions can be assumed to be realizations of a homogeneous Poisson point process (PPP), the MSE of the reconstructed field can still be analytically characterized under rather ideal conditions [321]. The effects due to realistic impairments such as quantization errors, energy constraints, and limited capacity further complicate the analyses, but some insights on the accuracy of the reconstruction process can be still extracted [322,323]. Recent studies have started to investigate possible extensions to the case of inhomogeneous sampling density [324], also considering stochastic sampling processes different from the PPP [325], so as to include additional aspects such as possible clustering effects [326] and the repulsion between points in the process [327]. An interesting comparison analysis between the deterministic and random sampling schemes has been conducted in [328], showing that the former provides improved reconstruction performance mainly in the high-SNR regime, while in the presence of low SNRs, both schemes behave in essentially the same way. A recent work explicitly analyzed the problem of reconstructing an environmental phenomenon f(p,t) from a set of samples randomly collected by crowdsensing vehicles [216]. By taking into account also the presence of a WSN infrastructure gathering data at fixed locations, it is demonstrated that stochastic sampling via crowdsensing leads to significantly improved reconstruction accuracy, especially when the WSN provides insufficient sampling information. Robust approaches able to infer environmental parameters from non-homogeneous and distribution-free data opportunistically gathered by crowdsensing nodes have also been considered [329].

The increasing availability of large unstructured datasets containing several different environmental data is stimulating the adoption of fully data-driven approaches to infer the behavior of environmental phenomena. A preliminary work tried to represent such “big environmental data” using the theory of *graph signal processing* [330]. An exploratory analysis has been conducted in [331] for the specific case of urban air pollution data, showing how the latter could be represented as a high-dimensional and geometrically structured graph signal. The analysis has been conducted on a real dataset of PM2.5 and NO${}_{x}$ measurements from New York City, and the results demonstrated that interesting information such as the identification of the most polluted areas could be readily inferred. This pioneering work opened up a promising research direction in which methods from the emerging field of graph signal processing can be used to analyze the complex dynamics of environmental phenomena [332,333].

#### 5.2.2. Sampling and Reconstruction with a Priori Information

When additional information on the physical field f(p,t) is available, more tailored sampling and reconstruction approaches can be used to further lower the number of required samples.

**Deterministic A Priori Information:** In some more controlled environments, it is possible to gain a better understanding of the physical phenomenon and establish *deterministic* (either physical or chemical) laws that accurately relate the involved environmental variables in time and/or spatial domains. An environmental monitoring system can be viewed as a set of heterogeneous sensors measuring different environmental variables. Let us denote with $f_{1}\left(t\right),\ldots ,f_{M}\left(t\right)$ the *M* different environmental variables measured by the sensors, where we considered the 1D case only to ease the notation. Assuming that ${\left\{f_{m}\left(t\right)\right\}}_{m=1}^{M}$ represent different manifestations of the same environmental phenomenon f(p,t), they can be linked through some underlying analytical relationship. For instance, if the behavior of the functions ${\left\{f_{m}\left(t\right)\right\}}_{m=1}^{M}$ can be linked via a system of linear differential equations with constant coefficients, describing the physical correlations among them, an additional constraint can be exploited in the reconstruction process

(8) $$A\left(\omega \right){\left[F_{1}\left(\omega \right)\;\cdots \;F_{M}\left(\omega \right)\right]}^{T}$$

where $F_{m}\left(\omega \right)$ denotes the Fourier transform of the *m*-th function $f_{m}\left(t\right)$ and A(ω) is a known P×M matrix, with *P* number of differential equations in the linear system. Interestingly, if we assume that the functions have a limited bandwidth in the interval [−B,B], but sensors are able to gather measurements only at a fraction of the corresponding Nyquist sampling rate $f_{c}=2B/K$, with K>0 denoting the *undersampling factor*, a perfect reconstruction of the original functions ${\left\{f_{m}\left(t\right)\right\}}_{m=1}^{M}$ is still guaranteed if [334]

(9) $$K\leq \frac{M}{M-P}.$$

The inequality in (9) conveys a rather important message: being that the upper bound is an increasing function of *P*, which we recall is the number of equations describing the physical model in (8), the more prior information about the environmental phenomenon is available, the less samples are required to perfectly reconstruct it.

A number of papers investigated different declinations of such a general framework to tackle more specific environmental monitoring problems, based on the availability of suitable physical models. For instance, the authors of [335] considered an array of chemical sensors and proposed novel algorithms to detect and localize potential sources of polluting vapors based on a generalized likelihood ratio test (GLRT). Flick’s law of diffusion has been used to model the pollutants released by a single source, emitted with a constant rate and starting from an unknown time instant. The same idea has been then extended to the case in which the chemical sensors are moving instead of fixed [336] and to more general air and ground scenarios [337]. To accurately model the diffusion of chemical pollutants in arbitrary 2D environments, a finite-element (FE) method has been integrated in [338] to linearize the spatial and temporal derivatives of the original differential equations. In addition to the detection and estimation of a biochemical source, the space–time pollutant concentration diffusion and its future evolution have been estimated in [339] using a maximum likelihood algorithm, taking as reference scenarios a propagation through two different semi-infinite mediums. With the aim of representing realistic scenarios while keeping the required computational complexity tractable, in [340] the biochemical dispersion model considered so far has been suitably approximated using a Monte Carlo numerical approach, which decoupled the fluid simulation from the transportation phenomena using a Feynman–Kac representation. The obtained model was general enough to emulate additional random effects such as chemical reactions, temperature effects, and radioactive decaying. A Bayesian framework has been considered to solve the associated inverse problem and retrieve the position of the polluting source. Similarly, in [341] the same approximated physical model has been coupled with a sequential change detection approach to promptly identify the time of release of biochemical pollutants. The authors of [342] addressed the problem of sampling and reconstructing the time-varying atmospheric emissions produced by industrial smokestacks. The physical phenomenon has been modeled using a set of partial differential equations (PDE), and sufficient conditions to estimate the emission rates were provided. In [343], the Fukushima disaster that occurred in 2011 was investigated using the above framework, with the aim of inferring the amount of radioactive material released and its consequences. The proposed method leverages sparse regularization to solve the problem and estimate the plausible amount of released Xenon, using an atmospheric dispersion model to emulate the radioactive emission process.

**Stochastic A Priori Information:** Although the deterministic modeling approach may be suitable for some specific and controllable environmental contexts, it cannot be easily applied in the presence of more complex phenomena. For instance, the air pollution in a crowded urban area is linked to several unpredictable factors such as the traffic density, the weather conditions, the specific type and configuration of the buildings, just to name a few. For these contexts, finding an appropriate physical model able to correctly capture all the involved variables and their interdependencies is a very challenging task, if not impossible. *Stochastic processes* represent an elegant way to overcome the lack of a physical model, as they allow a more general representation of an environmental phenomena through the use of regression or interpolation techniques, operating on all the measured data. A specific class used in most environmental contexts is that of *Gaussian Processes* (GPs), which are powerful tools for non-parametric regression able to provide an estimate of the field f(p,t) together with a measure of the corresponding uncertainty [344].

To simplify the exposition, we omit the time variations of the physical phenomenon and consider the sole dependency on the position p, thus obtaining a *spatial field* f(p). The spatial field is sampled by a set of *N* sensors located over the target area at known positions $p_{1},\ldots ,p_{N}$, with each observation expressed as

(10) $$y_{n}=f\left(p_{n}\right)+\epsilon_{n}\;n=1,\ldots ,N$$

where $\epsilon_{n}$ represents the (additive) measurement noise component modeled as an i.i.d. Gaussian random variable with zero mean and variance $\sigma_{n}^{2}$. (It is worth noting that each sensor *n* can perform multiple measurements of the spatial field, say $L_{n}$. Without loss of generality, we consider the case of $L_{n}=1,\forall n$.) The reconstruction process then consists in using the set of measurements and associated positions $Y=\left\{(y_{n},p_{n})|n=1,\ldots ,N\right\}$ to estimate the value of the unknown spatial field f(·) at a set of *regression* positions $p_{1}^{R},\ldots ,p_{T}^{R}$. In principle, a regression position could either coincide with a measurement position or not. Using the common terminology of machine learning, in the former case we refer to such points as *training* points, in which sensors aim at improving their local estimate of $f(p_{n})$. Conversely, in the latter case they are called *testing* points, and the corresponding estimates of $f(p_{i}^{R})$ are the interpolation (or prediction) of the spatial field f(·) at new unseen points of the space. If the spatial field admits a parameterization $f\left(p\right)=f\left(p;\theta_{1},\ldots ,\theta_{P}\right)$ in terms of a finite set of hyperparameters ${\left\{\theta_{p}\right\}}_{p=1}^{P}$, the latter can be inferred from the measurements set Y by using parametric regression techniques. As anticipated, in more complex environmental monitoring tasks, no exact or accurate parametric models for f(p) are available. A more convenient way to handle such contexts is to assume that the spatial field f(p) is a sample from a GP

(11) $$F\left(p\right)∼\mathrm{GP}\left(\mu \left(p\right),K\left(p,p^{\prime }\right)\right)$$

with mean function μ(p) and covariance function $K(p,p^{\prime })$. Customary covariance functions, also called *kernel* functions, include the Matern functions, the squared exponential functions, and the neural network functions, among many others. In other words, a GP imposes a multivariate Gaussian distribution over the space of functions that maps the sampling locations to the corresponding noisy measurements. It is not difficult to show that, since the joint distribution of the spatial field values measured at the training and test locations is Gaussian, so is the conditional distribution of $f(p_{i}^{R})$ at a generic test position $p_{i}^{R}$, given the measurements at the training positions $y={\left[y_{1}\;\cdots \;y_{N}\right]}^{T}$. Therefore, given y, it is possible to infer the distribution of the spatial field at a given test location $p_{i}^{R}$ as

(12) $$f\left(p_{i}^{R}|y\right)∼N\left(\mu_{i}\left(p_{i}^{R};y\right),\sigma_{i}^{2}\left(p_{i}^{R};y\right)\right)$$

where the conditional mean and variance are given by

(13) $$\begin{array}{cc} \mu_{i}\left(p_{i}^{R};y\right) & =E\left[F\left(p_{i}^{R}\right)|y\right]=\mu \left(p_{i}^{R}\right)+k^{T}{\left(K+\sigma_{n}^{2}I_{N}\right)}^{-1}\left(y-\mu \left(p_{i}^{R}\right)\right)\\ \sigma_{i}^{2}\left(p_{i}^{R};y\right) & =k\left(p_{i}^{R},p_{i}^{R}\right)-k^{T}{\left(K+\sigma_{n}^{2}I_{N}\right)}^{-1}k \end{array}$$

with E[·] denoting the expectation operator, $I_{N}$ the N×N identity matrix, k an N×1 dimensional vector whose *n*-th entry is the cross-covariance $k(p_{i}^{R},p_{n})$ between the test location and the *n*-th training location, and K the N×N covariance matrix of the training locations whose (n,j)-th element is given by $k(p_{n},p_{j})$, with n,j=1,…,N.

The probabilistic framework in (13) allows us to reconstruct an estimate of the overall spatial field f(p) by optimally combining all the measurements gathered by sensors in the monitoring system. Despite its elegant formulation, there are a number of aspects that need to be considered and some issues that should be carefully handled. For instance, the inversion of the N×N Gram matrix $\left(K+\sigma_{n}^{2}I_{N}\right)$ appearing in (13) can become computationally demanding as the number of sensors *N* increases. To overcome such a drawback, some works have proposed to approximate the Gram matrix with lower dimensional matrices acting as surrogates [345], or to exploit the possible regular displacement of sensors to implement a fast matrix inversion based on FFT approaches [346]. Other approaches have investigated the possibility of converting the covariance functions into infinite-dimensional stochastic differential equations [347]. In doing so, an approximation of the covariance function can be obtained in terms of an infinite-dimensional state-space model, where each element of the state coincides with a different order derivative of the original GP. As the solution of this approximate representation is a Markovian process, more efficient approaches such as the Kalman filter or its variants, possibly combined with a smoother one such as the Rauch–Tung–Striebel, can be used to compute the predictive distribution in (13) with a reduced (linear) complexity [348]. To cope with the more challenging case of large-scale monitoring based on crowdsensing, where the number of involved sensing nodes can be significantly higher, a suitably designed combination of GP and state-space models has been recently proposed in [349]. Interestingly, the proposed resolution method allows to compute (13) with a complexity that does not increase with the number of sensors *N*.

Besides computational aspects, a plethora of work has been devoted to the use of GP processes for environmental field estimation. For instance, the authors of [350] proposed a general modeling approach based on the Gaussian Markov random field, a specific category of GP, with the aim of inferring the behavior of a number of non-stationary environmental phenomena. An algorithm called the spatial best linear unbiased estimator has been derived in [351] to estimate standard spatial Gaussian fields, taking into account also the presence of non-idealities such as observation and quantization errors due to communications over the wireless channel. To infer information about environmental phenomena modeled by time-varying spatial GPs, a low-complexity regression method is presented in [352], which exploits only a limited set of measurements collected by a set of mobile sensor nodes (either terrestrial or aerial) with limited processing and storage capabilities. A Bayesian estimation framework has been applied in [353] to design algorithms able to compute the predictive distributions in (13) with a constant computation time, irrespective of the total number of measurements to be processed. In [354], a flexible generalization of a GP is proposed to estimate a spatial field by means of an empirical Bayes approach. Specifically, the main idea is to express the mean function μ(·) of the GP by means of general parametric equations. Such a reparameterization can be used to encode any a priori knowledge of the physics generating the spatial field, as well as any data-driven information. The authors of [355] framed the GP regression problem under an extended Bayesian optimization framework and solved the additional problem of accurately choosing the best measurement locations that maximize the regression accuracy while taking into account the distance traveled by mobile sensor nodes.

The average number of samples required to correctly infer the behavior of an environmental field can be further lowered if it admits a sparse representation on some basis [356]. In these contexts, *compressed sensing* techniques operating on infinite-dimensional and time-varying vectors represent a useful tool to guarantee a reconstruction up to a limited error in terms of MSE [357,358]. Although promising, such techniques are based on the underlying assumption that the environmental phenomenon possesses the SI property, or at least belongs to a space of generalized SI signals (combination of SI subspaces) [359]. Moreover, having a basis function representation for an environmental phenomenon means that the latter can be constrained to lie in a space having a fixed topology, which may not be often the case [360]. For the specific case of crowdsensing or UAV-based monitoring, distributed compressive sensing techniques can be employed to further lower the complexity of the reconstruction process by leveraging spatial and temporal correlations among data [361,362] and also when processing vectors of heterogeneous environmental measurements [220].

### 5.3. Environmental Monitoring Based on Hyperspectral Image and Signal Processing

Over the past decades, hyperspectral imaging has been affirmed as one of the leading technologies for a variety of environmental monitoring applications in different marine, land, and aerial contexts. Initially developed for remote sensing applications based on satellite and airborne systems, hyperspectral imaging has nowadays reached an unprecedented level of spatial, temporal, and spectral resolution, opening the door for new environmental monitoring applications requiring a very fine and detailed analysis of materials and their physical properties. Thanks to the rapid evolution of integrated sensor technologies, lightweight hyperspectral cameras can be easily carried by UAV platforms. Some new exciting applications of hyperspectral sensors recently appeared also in the field of smartphone-based environmental monitoring. This emerging topic, known as *smartphone spectroscopy*, has the ambition of making most of the powerful features of hyperspectral imaging available also in our compact handheld devices [363,364]. Handling such an increasing number of environmental applications calls for advanced signal processing algorithms able to cope with the complex process of extracting relevant information from hyperspectral data [365]. Very high dimensionality, spectral mixing effects (either linear or nonlinear), and a rather complex measurement process are only few examples of the several challenges behind the hyperspectral technology [366]. The theoretical tools used to deal with such issues span different fields, from image and signal processing to statistical inference and even the more recent machine learning and deep learning techniques.

#### 5.3.1. Hyperspectral Image Acquisition and Representation

In a nutshell, hyperspectral sensors aim at measuring the electromagnetic reflection, absorption and emission characteristics of a given material and to classify it based on its chemical and physical composition. The basic principle consists in acquiring the radiation generated by each object, represented by a set of pixels in a given scene, over a spectrum of different frequencies (i.e., different wavelengths). Collecting tens or hundreds of spectral channels allows to construct a *spectral signature* of the object of interest, which can be then used to recognize the nature of its constituent materials. Commercial hyperspectral cameras acquire data over a portion of the electromagnetic spectrum ranging from the visible region (from 0.4 to 0.7μm) to the short-wave (below 2.5 μm) and mid-wave infrared regions (below 5 μm), and even up to the long-wave infrared region (below 14 μm), with a sampling step between 6 and 10 nm. Given its three-dimensional nature, a hyperspectral image is usually referred to as *hyperspectral data cube* or *hypercube* and can be mathematically defined as a tensor $X\in R^{H\times W\times d}$, with *H* and *W* denoting the spatial size of the data and *d* the number of spectral channels (or bands). Notice that, due to the high spectral sampling frequency, all the *d* channels are highly correlated and the useful spectral information typically resides in a lower-dimensional manifold of the spectrum. The hypercube X is typically unfolded in two alternative ways before processing:

- As a set of d×1 vectors in the spectral dimension $x_{j}$, j=1,…,HW, with each $x_{j}$ representative of the *j*-th pixel in the image.
- As a set of H×W matrices in the spatial dimension $X_{i}$, i=1,…,d, with each $X_{i}$ a grey scale image containing all the pixels at the *i*-th spectral band.

**Figure 7:**
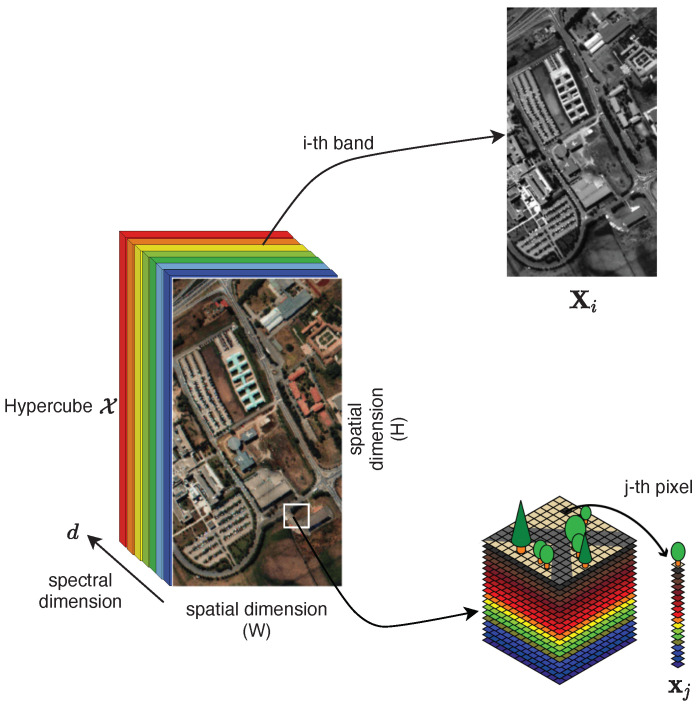
Two alternative unfoldings of the hypercube X along the spatial or spectral dimensions.

The first representation is useful when the different objects in the scene can be better separated in the spectral domain, owing to the principle that similar materials likely share similar spectral vectors $x_{j}$. Conversely, the second representation is more adequate when the objects’ similarities are more evident in the spatial dimension; hence, neighboring pixels can be clustered based on their correlations and different materials can be easily recognized through image segmentation techniques. We report a pictorial example of a hyperspectral image and its possible representations in Figure 7. The literature on hyperspectral imaging and signal processing is quite vast and covers a lot of different topics. In the following, we review three main categories of processing techniques, namely *classification*, *spectral unmixing*, and *change detection*, which are more frequently used in the environmental monitoring literature. We refer the interested reader to [367,368,369] and to the references therein for a more comprehensive overview of hyperspectral data analysis techniques.

#### 5.3.2. Hyperspectral Image Classification

Hyperspectral image classification is among the most active areas of research in the field of environmental monitoring [370]. An uncountable number of monitoring tasks including the identification of industrial discharges (solid and liquid residues, radioactive waste, gas emissions), the littering on coastal and sea surfaces, the outbreaking of wildfires, and many other land degradation phenomena can be accomplished by using hyperspectral classification algorithms. Given an observed hypercube X, the main goal of a classification algorithm is to associate each spectral vector $x_{j}$ with a specific class (or label) that identifies its nature. Individual classes or labels should try to uniquely represent a given type of material or land cover (e.g., distinguishing trees from soil, streets and buildings) and are usually defined by specifying some kind of similarity measure in the considered feature space [371]. Therefore, classification techniques are based on the underlying assumption that the spatial resolution of hyperspectral sensors is high enough to guarantee that each pixel in the image is characterized by a single dominant spectral signature (called *pure pixels*). When, conversely, images contain pixels whose spectral signatures are a mix of different components, spectral unmixing approaches need to be adopted, as will be discussed in the next section. Two subsequent steps are typically involved in the classification of a hyperspectral image:

- First, a *dimensionality reduction* is performed on the hypercube X to remove the redundant spectral information and keep only the most informative components, thus avoiding the curse of dimensionality and, at the same time, preserving the limited storage space available on UAV platforms [372].
- In a second step, a specific classifier is trained based on a chosen design strategy and used to label each spectral vector.

The dimensionality reduction step includes a feature selection and a feature extraction phase [373]. Given a set of initial features $F=\{F_{1},\ldots ,F_{d}\}$, *feature selection* aims at retaining only a small subset of them, denoted by $S=\{S_{1},\ldots ,S_{D}\}$, according to a suitable criterion that tries to preserve the classification performance, with D≤d and S⊆F [374]. On the other hand, the *feature extraction* phase aims at finding a suitable linear or nonlinear transformation $f:R^{d}\mapsto R^{D}$ that maps each higher-dimensional d×1 spectral vector $x_{j}$ into a corresponding reduced *D*-dimensional vector $z_{j}=f\left(x_{j}\right)$, j=1,…,HW, while trying to preserve the maximum amount of initial information in this new reduced feature space [375]. Based on whether it is possible to label each individual class or not, dimensionality reduction methods can be grouped into supervised, semi-supervised, and unsupervised approaches. Examples of supervised methods are the linear discriminant analysis (LDA) [376], the nonparametric weighted feature extraction (NWFE) [377], the mutual information [378], and their variants. Semi-supervised methods, used when only a very limited number of labels are available, include graph-based learning [379], cotraining [380], and transductive SVM [381], while common unsupervised methods are principal component analysis (PCA) [382], independent component analysis (ICA) [383], minimum noise fraction (MNF) [384], and their nonlinear versions.

The design of a specific classifier in the second step is conducted considering either spectral-only information or by jointly exploiting spectral and spatial dependencies. To simplify the design, approaches in the former group neglect the spatial correlations existing among pixels and treat the hypercube X as a set of independent spectral vectors $x_{j}$. As the identification of potential environmental risks (e.g., illegal landfills, abusive buildings) typically triggers a chain of legal and prosecutorial actions, an accurate classification of objects appearing in the scene is of utmost importance. This makes supervised spectral approaches largely preferable over both semi-supervised and unsupervised ones for environmental tasks. Unfortunately, in practical scenarios, the amount of training samples is often insufficient to allow a satisfactory training of supervised classifiers. Thus, handling high-dimensional data using only a limited amount of training samples represents one of the main challenges in the classification of environmental hyperspectral images. In addition to more traditional supervised approaches (e.g., SVM, random forest, back-propagation neural networks) [385], deep learning methods are also emerging as valid tools to learn high-level features that are inherently more robust against the non-idealities of hyperspectral images [386,387].

Considering that objects appearing in an environmental scene are usually larger than the dimension of a single pixel, neighboring pixels are inherently correlated in the spatial dimension. Such correlations provide additional information on the shape of the objects and can be thus fused with the spectral information to obtain improved classification performance [371]. Two main strategies can be adopted to infer the spatial information in the hypercube: a first family of approaches, called crisp neighborhood, extracts spatial information by considering only a restricted number of adjacent pixels and using a probabilistic model (such as the Markov random fields) to describe correlations as a two-dimensional stochastic process defined over a lattice of discrete points [388]. The second family of approaches, known as adaptive neighborhood, tries to extend the number of involved pixels by using image segmentation algorithms [389]. The spatial and spectral information are then fused by using composite kernel functions such as the generalized composite kernel (GCK) [390] or multiple-kernel learning [391].

Irrespective of whether spectral-only or combined spatial–spectral information is used for classification purposes, from the surveyed literature we can conclude that a general classifier that can be seamlessly used in all the diverse environmental contexts and still provide the best performance in terms of accuracy does not exist. To be effective, a hyperspectral image classifier should be designed by explicitly taking into account the different training and testing data at hand, integrating whenever possible also some a priori knowledge of the considered application domain.

#### 5.3.3. Hyperspectral Unmixing

The classification methods reviewed so far assumed that each spectral vector $x_{j}$ is characterized by a single dominant spectral signature. In realistic acquisition scenarios, sensing nodes (e.g., UAV platforms) are subject to a number of mechanical effects (e.g., vibrations, wind, adverse atmospheric conditions, and other effects related to the dynamics) that cause a destabilization of the hyperspectral sensor. As a consequence, the spectral content of pixels in the recorded hypercube X could be a mixture of radiations generated by many materials appearing at the same location covered by each pixel. Mixing effects can be also experienced when the hyperspectral sensor is perfectly stabilized but its spatial resolution is so high that intimate mixing phenomena spuriously appear. The latter case explains the well-known trade-off between spatial and spectral resolution in hyperspectral imaging. In all these cases, it is no longer possible to use the spectral signature of each pixel as discriminant to infer information about the materials present in the scene. To overcome such issues and restore the high classification performance obtained in the case of pure pixels, hyperspectral unmixing algorithms, also called blind source separation methods, need to be adopted. Basically, they try to exploit the rich spectral resolution intrinsinc in X to unmix the spectral contents of each pixel.

Hyperspectral unmixing has been widely investigated in the literature, and a number of methods have been proposed to mitigate or completely remove mixing effects under different operating conditions [392,393]. In the more specific environmental monitoring contexts, *linear mixture models* (LMMs) gained more attention due to their simplicity and mathematical tractability. Such models assume that mixing effects occur at a macroscopic scale and that each radiated signal is associated to a single material in the scene. The radiations emitted by the materials are therefore almost separated but, due to the limited spatial resolution of the hyperspectral sensor, appear as linearly superimposed in the recorded hypercube X. This is tantamount to modeling each measured spectral vector $x_{j}$ as the sum of *P* different spectral contributions as

(14) $$x_{j}=\sum_{p=1}^{P}\alpha_{j}^{p}m_{j}^{p}$$

with $m_{j}^{p}\in R^{d\times 1}$ denoting the spectral signature of the *p*-th material, known as *endmember*, while $\alpha_{j}^{p}$ quantifies the percentage of the *p*-th material in the *j*-th pixel, known as *fractional abundance*. Being that $\alpha_{j}^{p}$s are fractional values, they satisfy the abundance non-negativity constraint $\alpha_{j}^{p}\geq 0$, and their individual values are constrained by $\sum_{p=1}^{P}\alpha_{j}^{p}=1,\forall j$ (abundance sum constraint). Based on (14), the hyperspectral unmixing problem can be then recast as the problem of estimating the *P* endmembers $m_{j}^{p}$ and their associated fractional abundances $\alpha_{j}^{p}$, for each j=1,…,HW. Denote with $X=\left[x_{1}\;\cdots \;x_{HW}\right]\in R^{d\times HW}$ the matrix of hyperspectral data obtained by arranging all the spectral vectors in columns and, similarly, with $M=[m_{1}\;\cdots \;m_{HW}]$ the matrix containing all the endmember vectors $m_{j}={\left[m_{j}^{1}\;\cdots \;m_{j}^{P}\right]}^{T}$. Defining $A=[\alpha_{1}\;\cdots \;\alpha_{HW}]$ as the abundance matrix whose columns are the fractional abundance vectors of each pixel $\alpha_{j}={\left[\alpha_{j}^{1}\;\cdots \;\alpha_{j}^{P}\right]}^{T}$, the linear hyspectral unmixing problem can be thus formulated as

(15) $$\min_{A,M}{∥X-MA∥}_{F}\;\mathrm{subject}\;\mathrm{to}:\;A\geq 0,\;\;1_{P}^{T}A=1_{d}$$

where ${∥\cdot ∥}_{F}$ is the Frobenius norm operator and, with a slight abuse of notation, the abundance non-negativity constraint A≥0 should be intended in an elementwise fashion. Notice that (15) admits a double interpretation in terms of matrix factorization or, alternatively, as a linear blind source separation problem [394]. The main linear unmixing strategies used to handle hyperspectral environmental data can be broadly grouped in two categories based on whether endmembers are assumed to include pure pixels—for which sparsity or geometry-based approaches can be considered [395]—or, conversely, some of the endmembers are assumed to be missing in some pixels, using in those cases volume-based approaches [396,397]. In the presence of highly mixed data, however, both the previous approaches tend to fail, and the unmixing problem needs to be solved under a statistical framework [398], possibly considering the additional use of spatial information to complement the spectral one [399]. Other related research problems include the estimation of the actual number of endmembers *P* [400] and the characterization of their space-time variations [401], also including the extension to non-linear mixing models [368].

#### 5.3.4. Hyperspectral Change Detection

Besides classifying the different materials present in a target area at a given time instant, hyperspectral imaging can be used to support the continuous monitoring of a geographical surface, with the aim of promptly detecting significant changes that can occur over time [402]. In most environmental contexts, such changes could be indicative of man-made actions (either intentional or accidental) or potential natural disasters that may compromise environmental health [403]. To formally define the change detection problem, let us assume the availability of two hyperspectral cubes $X_{1}$ and $X_{2}$ capturing the same geographical area at two different time instants $t_{1}$ and $t_{2}$, respectively. By computing the hyperdimensional difference pixel by pixel, we obtain a new image

(16) $$X_{D}=X_{2}-X_{1}$$

encoding the spatial and spectral changes that occurred in that particular area. Assume that $H=\{H_{0},H_{1}\}$ is the set of all possible classes in the difference image $X_{D}$, with $H_{0}$ being the no-change class and $H_{1}=\left\{H_{1,1},H_{1,2},\ldots ,H_{1,K}\right\}$ being the set of *K* classes representing the different types of changes that can occur. Then, the task of identifying a significant change can be formulated as either a *binary* or a *multiclass* change detection problem. In the former case, the process aims at separating the no-change hypothesis $H_{0}$ from any possibly changed hypothesis in $H_{1}$, with no distinction within the latter set [404]. Conversely, in the latter case, the goal is to identify pixels with substantial changes in $X_{D}$ and to recognize the specific classes in $H_{1}$ that they belong to [405]. The general process of detecting changes in the difference image $X_{D}$ is typically split in two main steps.

**Anomaly Detection:** First, an *anomaly detection* process is conducted over the whole image with the aim of identifying pixels whose spectral variations are significant in magnitude (or according to other metrics) with respect to the other adjacent pixels representing the background [406,407]. Compared to more traditional optical images, where abrupt changes tend to emerge frequently, in hyperspectral imaging changes are intrinsically more implicit and complex, calling for advanced change-detection methods able to capture even very subtle variations that do not easily stand out from the background [408]. In this first processing step, targets of interest are not yet outlined and anomaly detection is conducted without any a priori spectral information. The Reed-Xiaoli (RX) detector represents one of the most effective algorithms to identify anomalies in a huge variety of environmental hyperspectral data. It was derived from the GLRT under the conservative assumptions of targets with unknown spectral distribution, embedded in background clutter with unknown spectral covariance. Denoting with $x_{t}$ a generic spectral vector extracted from the difference image $X_{D}$, to be tested against a possible anomaly, the RX algorithm consists in the following test [409]

(17) $${\left(x_{t}-{\hat{\mu }}_{b}\right)}^{T}{\hat{C}}_{b}^{-1}\left(x_{t}-{\hat{\mu }}_{b}\right)\begin{array}{c} \overset{\overset{}{>}}{\overset{<}{}} \end{array}\eta$$

where ${\hat{\mu }}_{b}$ and ${\hat{C}}_{b}^{-1}$ denote the estimated mean vector and covariance matrix of the background clutter, respectively, and η is a threshold to be set according to the desired probability of false alarm. Both $\mu_{b}$ and $C_{b}$ can be estimated globally on the whole hyperspectral image, using a multivariate Gaussian distribution to model the background clutter [410], resorting to linear subspaces [411], or through segmentation techniques inherited from optical image processing [412,413]. Alternatively, local estimation approaches based on sliding circular windows centered around a given pixel have been considered to obtain more accurate estimates of the background clutter, especially when some portion of $X_{D}$ can be excluded upfront [414]. In addition, several variations of the RX algorithm have been devised in the literature to achieve improved detection performance with possibly reduced false alarm rates [409,415].

**Target Detection:** In a second step, the identified anomalies are further verified through *target detection* methods to establish whether their spectral signatures can be associated to a target of interest (e.g., a pollutant material) or should be instead attributed to spurious variations caused by the natural clutter. Target detection methods leverage some a priori knowledge about the desired targets to infer their possible presence. Specifically, in the environmental monitoring literature, target materials are usually characterized by either a single spectral signature [416] or by specifying a signal subspace they belong to [417]. In the former case, the GLRT detector is the so-called spectral matched filter (SMF), whose decision statistic for a spectral vector $x_{t}$ under test is computed as [418]

(18) $$\mathrm{SMF}\left(x_{t}\right)=\frac{v^{T}{\hat{C}}^{-1}x_{t}}{v^{T}{\hat{C}}^{-1}v}$$

where v is the known target spectral signature and $\hat{C}$ is the estimated clutter covariance matrix. The SMF detector is derived by assuming that the background clutter follows a zero-mean Gaussian distribution N(0,C); the target is also Gaussian-distributed and shares the same statistics of the clutter but has a generally non-zero mean αv, with α the abundance factor encoding how strong the target presence is inside the pixel vector $x_{t}$. To overcome possible numerical issues arising in the computation of the decision statistic (18), the SMF detector can be modified by discarding eigenvectors whose associated eigenvalues fall below a predefined noise threshold [419], or by adding a regularization factor to $\hat{C}$ before its inversion [420].

When both target and natural clutter spectral signatures can be assumed to belong to two separated linear subspaces, the GLRT detector is represented by the subspace matched detector (MSD), having the following decision statistic

(19) $$\mathrm{MSD}\left(x_{t}\right)=\frac{x_{t}^{T}\left(I_{d}-P_{c}\right)x_{t}}{x_{t}^{T}\left(I_{d}-P_{v}\right)x_{t}}$$

where $P_{c}$ and $P_{v}$ represent the projection matrices onto the clutter-only and target plus clutter subspaces, respectively. By elaborating on the same idea, alternative detectors such as the adaptive subspace detector (ASD) [421] and the orthogonal subspace projection (OSP) [422] have been proposed in the literature. These three detectors are still considered the benchmarks for target detection in hyperspectral imaging.

## 6. Integrated Large-Scale Air–Ground Environmental Monitoring

The review of the individual WSN, UAV, and crowdsensing technologies outlined that, despite remarkable improvements in some specific application contexts compared to the use of legacy systems, the monitoring capabilities for most approaches proposed in the literature are still limited by either the insufficient information gathered by sensor nodes (incomplete or inaccurate) or by some intrinsic limitations of the adopted technologies (e.g., limited sensor autonomy, restricted operating areas). Nonetheless, the rapid impact of environmental changes on both the economy and health of the modern society signals the need for advanced monitoring systems able to operate on large geographical scales, and to provide integrated (air, land, sea) real-time and long-term analyses from a big amount of heterogeneous environmental data. A possible way to meet such requirements is to consider the design of *hybrid* monitoring systems that try to combine the benefits of more technologies.

### 6.1. Hybrid Environmental Monitoring Systems

A first step toward this idea is, for instance, the hybridization between the ground sensors of a WSN and the aerial UAV nodes, a topic that has started to attract attention in the environmental research community [52,423]. Complementing a fixed WSN with UAV nodes brings three main advantages: (i) a more extended and finer monitoring of the target area becomes possible thanks to the aerial inspection capabilities of UAVs, which enable a more focused and controlled analysis of the environmental phenomena, reducing the risk of losing potentially critical data; (ii) a better management of the energy consumption can be achieved by designing suitable interplay sensing strategies between WSN and UAV nodes which, by extending the autonomy of each individual sensor (either fixed or mobile), can prolong the whole system lifetime and ensure a long-term monitoring of the area of interest [424]; (iii) an increased flexibility of the monitoring system is obtained thanks to the versatility of UAV nodes, which can be easily converted to mobile base stations and used to extend the communication range between the WSN and the monitoring centers, or even used to support the physical deployment of WSN nodes [425]. When applied to the fields of air pollution or radioactivity monitoring, a hybrid WSN-UAV system can leverage its three-dimensional inspection capability to provide a much more accurate prediction of the propagation and evolution of pollutants over time [426,427], allowing a better assessment of their potential impact over a large geographical scale [428]. Hybrid WSN-UAV systems can also bridge the gaps existing in marine applications, where access to WSN nodes disseminated over the sea surface is often very difficult, or almost impractical in cases of underwater deployments [429]. In such contexts, the WSN can be employed for data collection (e.g., temperature, humidity, turbidity), while UAVs mainly act as aerial sinks for the periodic offloading of such data. An experimental test conducted in [430] demonstrated that, remarkably, a collaborative WSN-UAV monitoring system can achieve an extended operating range of about 5 km from the coast, while guaranteeing a bit rate of about 10 kbps. WSN and UAVs can actively cooperate to support authorities in the presence of natural disasters, from the initial provisioning phase up to disaster recovery [431]. Since disaster management applications usually operate in harsh environments, UAV nodes can be employed for a preliminary scanning of the target area, with the aim of identifying the best candidate zones to perform aerial and ground measurements [432]. Then, in a second step, WSN nodes equipped with proper sensing units are deployed and used to carry out more detailed measurements [433]. A key advantage of a hybrid WSN-UAV system is its capability to dynamically readapt to the priorities of the disaster scenario at hand, either in terms of safeguarding human lives or providing real-time analyses based on the intelligent fusion of all the available environmental data [53].

Almost along the same line, some recent work has started to consider a combination of WSN and crowdsensing as well as a combination of crowdsensing and UAV technologies. The former systems aim at increasing the too-coarse sampling capability of WSNs by integrating measurements opportunistically gathered by mobile crowdsensing nodes on a denser scale. From a spatio-temporal perspective, the two technologies reinforce each other: mobile crowdsensing can be largely exploited during the daytime, when citizens carry out their daily activities. In the meantime, WSN nodes can be set in sleep mode to preserve their energy. Conversely, during the night time, when the number of crowdsensing nodes is typically scarce, the hybrid system can schedule WSN nodes to perform data collection. Hybrid WSN-crowdsensing monitoring systems have found some applications in the field of air quality monitoring to face the significant spatial variability of particulate matters [54,434]. A prototype system based on low-cost, off-the-shelf hardware mounted on both fixed (WSN) and mobile (crowdsensing) nodes has been recently developed in [435]. Preliminary results from six months of data demonstrated that the system can reliably reveal areas with exceeding levels of PM2.5 and PM10 and, interestingly, can guarantee a high sampling rate of about one measurement per second. Similarly, hybrid crowdsensing–UAV monitoring systems have been conceived with the idea of broadening the sensing horizon of mobile crowdsensing nodes, especially when operating in areas that could be hardly reached by terrestrial vehicles (e.g., flooded areas, forest areas) [436,437]. Additionally, in this case, the few available prototypes of collaborative crowdsensing–UAV systems have been mainly employed for air-quality monitoring [438], also considering the reuse of UAV platforms dedicated to package delivery [439].

### 6.2. Combining WSN/UAV/Crowdsensing and Advanced Signal Processing

Despite that significant work has been devoted to the application of each *specific technology* to the diverse environmental contexts, as illustrated in Section 2, Section 3 and Section 4, the interlinking between different types of technologies has only recently appeared in the literature and its potential has not yet been fully explored. More specifically, most of the hybrid systems discussed in Section 6.1 are often restricted to quite limited areas and are intended only for a few specific types of analysis (e.g., only marine pollution or only air pollution). Furthermore, the design approaches mainly focus on aspects related to the degree of *reactivity* of the monitoring system, i.e., the ability of the proposed system to respond promptly to the occurrence of critical events such as, for instance, large wildfires or spills of pollutants into the sea. However, less attention has been devoted to the possibility of using, in a complementary way, *proactive* approaches that allow for identifying potential high-risk areas and to implement preventive actions in advance, aimed at reducing the probability of events with a high environmental impact. Overall, none of the available systems is able to ensure an *integrated* monitoring of the environment in all its aspects (air, land, and water), while offering a cost-effective and scalable solution to support a *large-scale* coverage. In this respect, we believe that the integration of the three different monitoring technologies (WSN/UAV/Crowdsensing), coupled with the use of advanced signal processing techniques such as those presented in Section 5, will be at the basis of future monitoring systems, igniting a new era of *ubiquitous environmental monitoring*.

**Figure 8:**
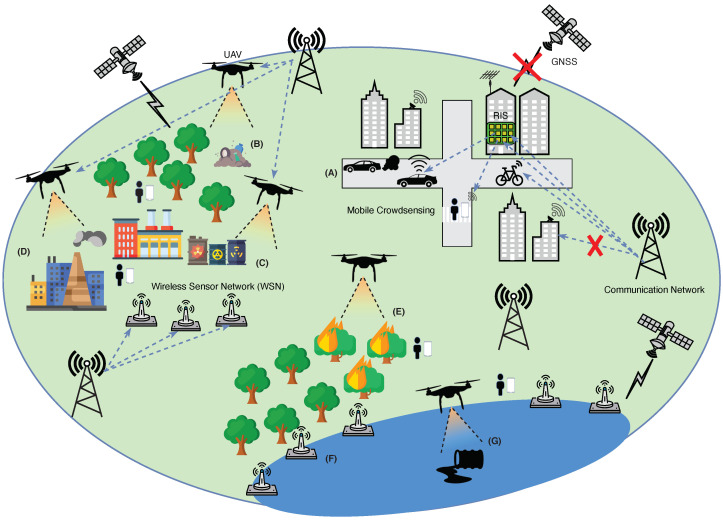
Overview of potential uses of WSN, UAV, and crowdsensing inspection capabilities to enable integrated and large-scale environmental monitoring.

In Figure 8, we depict the main application contexts of a unified framework for integrated large-scale environmental monitoring, with the aim of illustrating tasks for which a combined use of air–ground technologies is possible, and those for which WSN, crowdsensing, and UAV are alternative or complementary. More specifically, scenario (A) represents a typical urban environment characterized by the presence of several potential sources of pollution: on the one hand, there are a number of electromagnetic sources such as antennas installed on the roofs and mobile devices transmitting in the area; on the other hand, a number of vehicles passing through the intersection may contribute to the potential increase in air pollution levels (due to engine exhausts) and noise pollution levels due to engine noise (e.g., heavy traffic, motorbikes, and large cars) or an improper use of horns. In this application context, a set of mobile devices recruited as crowdsensing nodes provides important real-time information related to some key environmental parameters. This information can be processed through advanced signal processing algorithms (such as those discussed in Section 5) to constantly monitor the spectrum of radio and acoustic frequencies, quantifying the energy content of the detected signals and assessing the consequent impact on the environment. By combining this information with that deriving from the analysis of vehicle emissions, it is also possible to infer useful information to support applications for traffic control and road safety (e.g., to avoid frequent congestion on the main city backbones and to reduce the number of accidents). Scenario (B) is representative of a case of soil pollution caused by the illegal dumping of solid waste. In this case, one or more UAVs can be used to acquire images/videos and data from hyperspectral sensors. Such data can processed through hyperspectral signal processing techniques (see Section 5.3) to analyze the physical characteristics of the different materials and classify them according to their spectral signatures. Subsequently, the optical data can be used to validate and confirm the actual presence of waste in the surveyed area. Scenario (C) illustrates a case in which some industrial waste may have spilled into the soil. In order to cope with this scenario, the air–ground framework combines the use of UAV nodes with the sensing capabilities of a WSN. The latter, in fact, thanks to the availability of sensors dedicated to the analysis of chemical and biological substances, is able to identify any contamination of the subsoil that could not otherwise be revealed through the data acquired by UAVs. Similarly, scenario (D) relates to the emission of industrial smokes and can be addressed through the joint use of WSN and UAV nodes, and it is further enhanced by including the contribution from citizens. Specifically, air pollution measurements gathered by WSN nodes can be exploited to determine if the maximum tolerable thresholds in the area have been exceeded. When this happens, WSN data will be integrated with the optical and hyperspectral data acquired by the UAVs to locate the pollutant source, using information from crowdsensing nodes (e.g., photos) to support the identification process. Scenario (E) illustrates a typical case of forest fire, for which the sensing capabilities of a fleet of UAVs play a key role. On the one hand, the processing of hyperspectral data allows the early detection of a fire outbreak. In the event that a potential fire is identified, if the UAV nodes are also equipped with LIDAR sensors, these can be used to verify the correctness of the triggered event, thus reducing the number of false alarms and saving important emergency response resources. In addition to fire detection (reactive approach), data from optical and hyperspectral sensors can be combined with measurements from thermal sensors to identify high-risk areas (proactive approach). For instance, areas with a high density of dry vegetation and high temperatures may be subject to preventive actions aimed at avoiding the outbreak of fires. Scenario (F) represents a typical marine environment. In this context, a WSN can be deployed on the shore and/or on appropriate buoys located at strategic points of the sea. By fusing information from pressure, thermal, and biological sensors, it is possible to monitor important parameters (e.g., water turbidity level, presence of biological indicators) in real time. Moreover, the possible presence of pollutant spills in the sea shown in scenario (G) can be revealed by processing, in addition to the data from WSN nodes and acquired by UAVs as well as the explicit feedback coming from citizens. Overall, it is evident that the three air–ground monitoring technologies (WSN/UAV/crowdsensing) can be used in a synergistic manner to compensate for the limitations of each individual technology, motivating their joint use in future integrated and large-scale monitoring systems.

**Figure 9:**
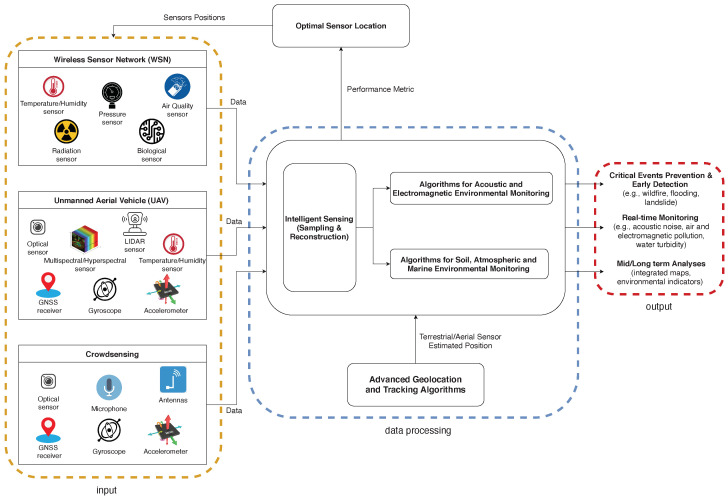
Main components of the proposed three-level monitoring architecture combining WSN/UAV/crowdsensing technologies and advanced signal processing.

The idea of creating a collaborative system made of fixed and mobile terrestrial nodes, as well as of aerial UAV platforms, is indeed not new. In recent years, we have been witnessing an impressive growth in the number of smart devices and services that need to be supported by existing communication networks. To fully manage the ever-increasing data demands generated by the several heterogeneous applications emerging under the IoT umbrella, architectures at the basis of next-generation 5G and beyond (6G) communication networks will integrate satellite, aerial, and terrestrial communications under the so-called *space–air–ground* network paradigm [440,441,442]. It is thus natural to look at environmental monitoring as a big and complex service that can be supported by such emerging architectures. For instance, in [443], it was shown that a combination of smartphones, sensors, and UAVs can effectively overcome the limitations of an unstable communication environment in the presence of a natural disaster. However, several other aspects besides communication should be taken into account to treat the topic in its entirety. Indeed, the considerable complexity of ensuring integrated large-scale environmental monitoring is also related to the great diversity among the air, land, and water contexts, to the huge variety of sensing platforms carrying heterogeneous sensors, and more generally to the specific characteristics of each individual monitoring task. In Figure 9, we identify the main components of a high-level architecture that leverage the different sensing capabilities available on the three levels made up of WSNs, crowdsensing, and UAVs, to enable large-scale integrated monitoring (atmospheric, marine, acoustic, electromagnetic, and soil) of the environment.

The basic idea consists in interpreting the whole ecosystem, including heterogeneous terrestrial (WSN/crowdsensing) and aerial (UAV) sensors, under a unified multi-agent and multi-system framework. The first fundamental block is represented by the input level, i.e., the set of all the heterogeneous environmental data collected by the various sensor nodes. It is important to note that the information flow generated by the input level is generally:

- *Asynchronous* since the actual availability of input data varies according to the sensing performed by the three different architectural levels (WSN/crowdsensing/UAV) at different time instants;
- *Non-uniform* as the measurements acquired by the various sensor nodes are linked to the specific application scenarios in which they operate and, therefore, are associated with areas not homogeneously distributed over the entire territory.

The second fundamental block of the proposed framework concerns the processing of collected data and is articulated in four parts:

(i) **Advanced Geolocation and Tracking:** A major transversal issue concerns the correct attribution of a geographic position information to environmental data gathered by ground-based (crowdsensing) and aerial (UAV) mobile sensor nodes. Specifically, information opportunistically obtained through crowdsensing is typically available on the basis of users’ mobility and requires innovative algorithms to be spatially contextualized (geo-referenced), especially when nodes operate in contexts where common satellite navigation systems (GPS) are inaccurate or completely unavailable (e.g., in dense urban environments). Similarly, data from UAVs must also be accurately localized and tracked over time. In particular, advanced algorithms are required to extend the capabilities of the on-board GNSS receiver so as to handle the high manoeuvring speeds of such platforms and to guarantee their accurate localization even when operating in hostile or hardly accessible environments (e.g., forests, caves). The output of this module consists of *position estimates* at the time instants corresponding to mobile sensors measurements;
(ii) **Intelligent Sensing:** The availability of statistically significant indicators is of utmost importance for a correct analysis and mapping of the different pollution phenomena. To this end, a key role is played by signal processing techniques involving the statistical modeling of measurement and sampling processes, whose main goal is to infer the main parameters of a given pollution phenomenon, modeled through either a deterministic (physical) or a stochastic spatio-temporal model, starting from a partial set of observed samples. Using the position estimates produced by the data geolocation module, the collected measurements can be spatially correlated and appropriately combined through *data-fusion* approaches in order to enable an integrated monitoring of the parameters of interest;
(iii) **Acoustic and Electromagnetic Environmental Monitoring:** Another important aspect concerns the processing of acoustic and electromagnetic measurements coming from single sensors or sensor arrays (multiple antennas/microphones), with the aim of both identifying possible sources of pollution and monitoring a set of environmental parameters of interest. The processing algorithms to be considered in this field are mainly based on theoretical tools such as *detection* and *spectrum sensing*. Effective solutions should be able to provide a continuous monitoring of the frequency spectrum (radio and acoustic) in order to identify and classify the various electromagnetic sources, quantifying the energy content of the signals detected and assessing their consequent impact on the environment;
(iv) **Soil, Atmospheric, and Marine Environmental Monitoring:** To complement the previous module, statistical methods to detect and estimate the dispersion of a specific (air, land, or sea) pollutant by adopting analytical diffusion models should also be considered. Such approaches can be useful to determine the spatio-temporal concentration distribution of a specific pollutant and to predict its future evolution. Multispectral/hyperspectral imaging represents another valuable source of information. Through the processing of such data, it is possible to analyze the physical characteristics of the different materials present in a target area and to recognize them on the basis of their *spectral signatures*, using both *classification* or *spectral unmixing* tools. Effective solutions should be able to identify the possible presence of pollutants dispersed on the land (e.g., spills in the sea, illegal dumps of wastes, …) or to promptly reveal the onset of critical events such as wildfires and floods.

Another important aspect is the choice of the most informative sites where sensor nodes should perform the sensing campaign. When sensor positions can be flexibly adapted to the specific monitoring task at hand (e.g., deployment of a WSN in a fully controllable environment), signal processing techniques such as those discussed in Section 5.1 should be additionally considered to make better use of sensing resources and further improve the estimation and analyses of environmental phenomena. To support the emerging needs in terms of seamless environmental monitoring, the last block of the framework should enable:

- The possibility of outlining *proactive* interventions aimed at reducing or completely avoiding the occurrence of environmental disasters. When this is not possible, a prompt detection of any natural hazard in its early stage must be anyway guaranteed, providing useful information that can be used by the competent authorities to limit the potential damages;
- A *real-time* monitoring of a selected set of indicators that reflects the state of environmental health. Necessary elements include the levels of acoustic noise, the levels of air pollutants (PM2.5 and PM10), the levels of radiation, and the levels of water turbidity;
- *Mid and long-term* analyses based on the big environmental data collected and stored over time. Such historical information can be used to continuously update the prediction models—used, for instance, by GISs—and to maintain accurate integrated maps of the main environmental phenomena over a large geographical scale.

### 6.3. Future Perspectives

**Figure 10:**
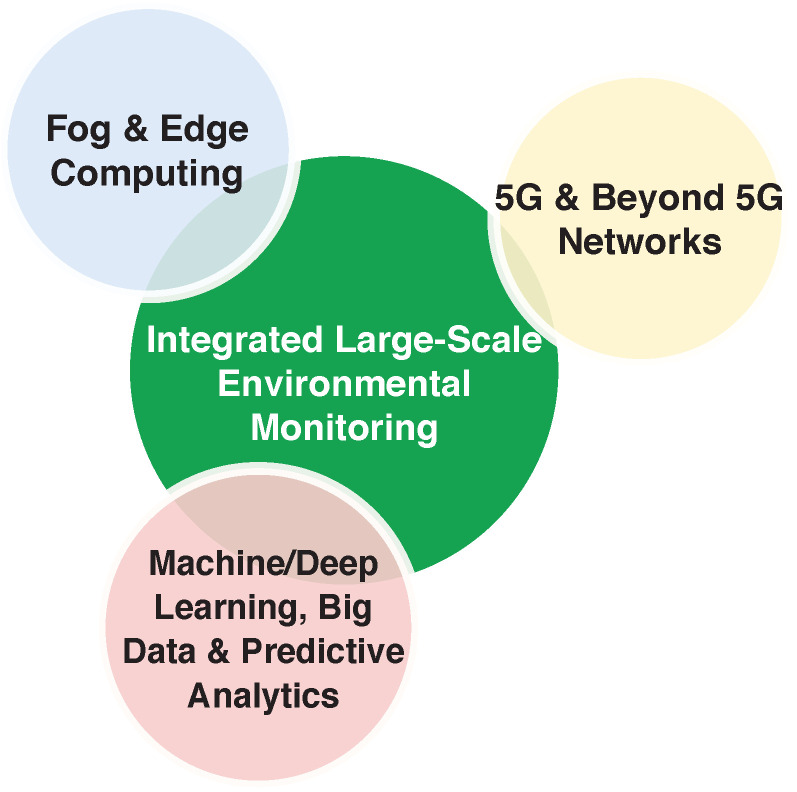
Research areas playing a key role in future integrated large-scale environmental monitoring systems.

In summary, the high-level idea presented so far is to accomplish environmental tasks by *adaptively* exploiting all the available information in each operational context, using advanced signal processing as a key ingredient to obtain low cost and scalability, both prerequisites to enable integrated and large-scale monitoring. On the one hand, this opens up unprecedented opportunities to design novel environmental monitoring systems able to extend the capabilities of the existing systems. On the other hand, it faces new challenges related to the interaction of different systems and technologies, which results in an overlap of several problems such as estimation and information fusion, localization and tracking, statistical analysis of the heterogeneous environmental data, and the design of proper sensing strategies. Therefore, there still exists a noticeable gap to be filled in order to ensure a successful development of future environmental monitoring systems based on air–ground (WSN/UAV/crowdsensing) sensors. In our view, the intrinsic interdisciplinarity of the topic requires that advances in different research areas are jointly achieved in the next years. More specifically, we identified the following three main areas, as shown in Figure 10:

- *Machine/deep learning, big data, and predictive analytics:* According to a recent report by Cisco, the sole environmental data sensed by WSNs and crowdsensing nodes in urban environments are expected to increase up to 5 ZB per year by 2021 [444]. Such heterogeneous data are characterized by a large variability and large volumes and exhibit significantly different accuracy owing to the different types of sensors. In this respect, more advanced big data analytics need to be devised to extract meaningful information from a plethora of non-uniform raw environmental data, leveraging the joint processing power of both fixed and mobile nodes [445] and treating the whole ecosystem made of air and ground sensors as a smart and interconnected large-scale community [446], enabling the so-called *smart environmental monitoring* [37]. Machine/deep learning techniques represent another fundamental tool to manage large volumes of heterogeneous data for which analytical models are not often available [447]. Besides being used to enhance the performance of specific tasks such as, for instance, classification in hyperspectral imaging, such techniques can be extended also to support the design of optimal sensing strategies, with the aim of striking a sustainable balance between sensing quality and cost involved in the sensing campaign [448]. With the increasing availability of large environmental datasets, deep learning algorithms able to infer representations of data at different levels of abstraction will be also necessary [449]. As a fundamental enabler for most monitoring tasks, predictive analytics are required to combine big data and machine learning/deep learning and predict future evolution and impacts of environmental phenomena using both data-driven and model-based approaches [450].
- *Fog computing and mobile edge computing:* The potentially very high number of devices available when joining air and ground sensing capabilities over large geographical areas can seriously challenge most of the existing computing paradigms (e.g., cloud). A paradigm shift moving the intelligence closer to the sensing devices can represent a win–win strategy to guarantee a seamless environmental monitoring service while also fulfilling important requirements such as low-latency, availability of high dedicated bandwidths for data transfer, and context awareness for allocating sensing tasks and full support of node mobility [451]. *Fog computing* can suit such needs by making some of its multiple architectural layers available in the proximity of the sensing devices. Each layer is conceived with a significant processing, communication, and storage capability and is meant to support sensing nodes in performing preliminary local tasks [452]. Elaborating on the same idea, *mobile edge computing* aims at injecting application-oriented capabilities directly in the core of network operators, possibly providing most of the network and processing services at a one-hop distance from the sensing devices [453]. By working on top of secured peer-to-peer networks, sensing nodes can safely share the collected environmental data, whereas monitoring centers can have full control of the flow of sensed data [454].
- *5G and beyond 5G networks:* The network infrastructure must be able to support different communication requirements according to the specific monitoring tasks and the involved sensing devices. For instance, UAV nodes require high availability of wireless links in order to be remotely piloted/controlled. Moreover, the periodic offloading of data from UAVs could involve several tens of GB, especially in the presence of data acquired by optical or hyperspectral sensors. On the other hand, crowdsensing nodes typically operate in urban environments, where signal obstruction phenomena (e.g., attenuation effects, multipath) are frequent owing to the presence of obstacles such as buildings, tunnels, and vehicles [455,456]. Supporting high data rates, ultra-reliable low-latency communications, and massive connectivity are among the main objectives of the emerging 5G cellular networks [457]. By exploiting the presence of multiple directional antennas at both the transmit and receive sides under the multiple-input multiple-output (MIMO) paradigm, 5G systems will guarantee more efficient wireless communications thanks to the beamforming technology, while simultaneously supporting critical services such as localization and context awareness [458,459]. In addition, communicating at *mmWave* frequencies allows for benefiting from higher bandwidths and, in turn, from higher data rates. Further improvements can be obtained by considering the use of the emerging *reconfigurable intelligent surfaces (RIS)*. Such a technology, which will be at the basis of future 6G networks, allows wireless communications to evolve toward a new reality where the propagation environment can be re-engineered, i.e., dynamically programmed and reconfigured to adapt to the surrounding environment [460]. These artificial surfaces, made of electromagnetic material, can modify the propagation of radio waves (by acting the way that they interact with surrounding objects—see Figure 8, scenario A), thus attenuating the negative effects of propagation (path loss, multipath fading) and allowing for the establishment of a robust communication link even when the direct path between transmitter and receiver is severely obstructed. Notably, RISs are conceived as fully passive devices and as such represent a big promising step toward achieving pervasive but sustainable, reliable, and eco-friendly *green communications* [461,462].

## 7. Conclusions

The unprecedented environmental challenges faced by modern society call for the need of advanced solutions able to guarantee a pervasive and continuous monitoring of the environment in all its aspects (air, land, and water). With the increasing availability of WSN, UAV, and crowdsensing technologies, nowadays endorsed with significant sensing and processing capabilities, a new frontier of environmental monitoring empowered by a large number of low-cost devices and offering a denser and more accurate coverage of the territory has emerged. In this paper, we provided a systematic review of the main solutions proposed in the field of WSN, UAV, and crowdsensing monitoring, harmonizing the huge amount of work scattered across different research communities according to each specific application context (air, land, or sea). Based on such a classification, we highlighted the main benefits and open challenges of each individual technology. As a second main contribution, we analyzed the signal processing literature and conducted a detailed review of the most relevant methodologies applied in the field of environmental monitoring. Remarkably, it has been found that advanced signal processing plays a key role in a number of environmental tasks, from the choice of the optimal sites for sensor placement to the accurate reconstruction of physical phenomena and effective identification of polluted areas and natural hazards. We then identified the main components of a three-layer architecture that, by joining the ground and aerial sensing capabilities of WSN/crowdsensing and UAVs, can enable integrated and large-scale monitoring of the environment, leveraging advanced signal processing to promote cost-effectiveness and scalability. Finally, a perspective of future research directions has been provided, outlining that a synergy between different research areas is necessary to handle the intrinsic multi-disciplinarity of the topic.

## Figures and Tables

**Table 1:** Related survey papers on WSN/UAV/crowdsensing environmental monitoring.

Techn.	Title	Ref.	Main Content
Wireless Sensor Networks (WSNs)	Environmental Sensor Networks: A Revolution in the Earth System Science?	[34]	A review on technological evolution from legacy systems to WSNs
	Marine Environment Monitoring Using Wireless Sensor Networks: A Systematic Review	[35]	An overview of applications of WSNs to marine environmental monitoring
	Energy Efficient Solutions in Wireless Sensor Systems for Water Quality Monitoring: A Review	[36]	A review of applications of WSNs to water monitoring
	Advances in Smart Environment Monitoring Systems Using IoT and Sensors	[37]	A review on technological advancements in the development of modern WSNs
	Review of Wireless Acoustic Sensor Networks for Environmental Noise Monitoring in Smart Cities	[38]	A review of most relevant WSN-based approaches for acoustic noise monitoring
	Energy-Efficient Wireless Sensor Networks for Precision Agriculture: A Review	[39]	A review on recent applications of WSNs in precision agriculture research
Unmanned Aerial Vehicles (UAVs)	On the Use of Unmanned Aerial Systems for Environmental Monitoring	[40]	A survey on applications of UAVs in natural and agricultural ecosystem monitoring
	Current Practices in UAS-based Environmental Monitoring	[41]	A review of UAV-based environmental monitoring using passive sensors
	Hyperspectral Imaging: A Review on UAV-Based Sensors, Data Processing and Applications for Agriculture and Forestry	[42]	A review of UAV-based hyperspectral remote sensing for agriculture and forestry
	Unmanned Aerial Systems (UASs) for Environmental Monitoring: A Review with Applications in Coastal Habitats	[43]	A review of emerging applications of UAVs for mapping coastal habitats
	A Review on Early Forest Fire Detection Systems Using Optical Remote Sensing	[44]	A review on UAV-based optical remote sensing for early detection of forest fires
	Thermal Remote Sensing from UAVs: A Review on Methods in Coastal Cliffs Prone to Landslides	[45]	A review on UAV-based thermal remote sensing for monitoring landslides
	A Review on Air Quality Measurement Using an Unmanned Aerial Vehicle	[46]	A review on the use of UAVs for air quality monitoring
Crowdsensing	A Survey on Mobile Crowdsensing Systems: Challenges, Solutions, and Opportunities	[47]	A survey on applications of crowdsensing for data collection in different contexts
	Sensors and Systems for Wearable Environmental Monitoring Toward IoT-Enabled Applications: A Review	[48]	An overview on the emerging wearable environmental monitoring systems
	On Enabling Mobile Crowd Sensing for Data Collection in Smart Agriculture: A Vision	[49]	A survey on the use of mobile crowdsensing for smart agriculture
	A Survey on Mobile Crowd-Sensing and Its Applications in the IoT Era	[50]	A survey on the use of smartphones’ built-in sensors and their applications
	Use of Social Media Data in Disaster Management: A Survey	[51]	A survey on methodologies that use social data crowdsensing for disaster management
WSN-UAV	A Survey of Collaborative UAV–WSN Systems for Efficient Monitoring	[52]	A survey on the joint use of WSN and UAV for efficient monitoring tasks
	Wireless Sensor Networks and Multi-UAV Systems for Natural Disaster Management	[53]	A review of the main applications involving WSNs and UAVs in disaster management
WSN Crowd.	Prospects of Distributed Wireless Sensor Networks for Urban Environmental Monitoring	[54]	A survey on the joint use of WSN and crowdsensing for urban pollution monitoring

**Table 2:** Typical sensors used in WSN-based environmental monitoring systems.

**Physical Environmental Parameters**		
**Type**	**Sensor Technology**	**Operational Range**
Temperature	thermal resistor, resistance temperature detector (RTD)	−60 to +90 °C
Pressure	integrated electromechanical, piezoresistive	700–1100 mbar
Turbidity	nephelometric	0–4000 NTU
Air Flow	thermal anemometric, mechanical	0–80 m/s
Radiation	radiation thermocouples, photodiode	0–1500 W/m${}^{2}$
**Chemical Environmental Parameters**		
**Type**	**Sensor Technology**	**Operational Range**
PM2.5/PM10	optical scattering, radiating particles, light detection	0–500 mg/m${}^{3}$
NO${}_{x}$	electrochemical, chemiluminescence	0.05–5 ppm
SO${}_{2}$	electrochemical, ultraviolet fluorescence	0.05–5 ppm
O${}_{2}$	chemiluminescence	0.01 mg/L–2000 mg/L
O${}_{3}$	ultraviolet photometry, chemiluminescence	0.05–5 ppm
CO	electrochemical, MOX	0.05–500 ppm
CO${}_{2}$	NDIR	0.1–5000 ppm
VOCs	mechanical resonator	1–1000 ppm
pH	electrochemical	0–15 pH

**Table 3:** Common UAV platforms for environmental monitoring.

UAV Type	Average Coverage	Main Characteristics
Fixed wing	greater than 20 km${}^{2}$	ability to survey large areas, higher velocity reduced startup time
Multirotor	from 5 km${}^{2}$ up to 30 km${}^{2}$	ability to hover, flexible and stable, low altitude and low speed inspection
Hybrid VTOL	in the order of 100 km${}^{2}$	ability to hover, survey very large areas, vertical take-off and landing capabilities

**Table 4:** Typical sensors used in UAV-based environmental monitoring systems.

Type	Sensor Technology	Main Applications
Optical Camera	optical RGB	aerial photogrammetry, detection, 3D modeling and reconstruction
Thermal	resistive bolometers, pyroelectric devices	thermography, heat mapping, water temperature, level of soil water
Multispectral	filtering, infrared and ultraviolet sensors	wildfire detection, soil classification, vegetation mapping, water analysis
Hyperspectral	modular spectrometer	wildfire detection, soil classification, materials analysis, water analysis environmental mapping
LIDAR	pulsed laser	3D mapping, wildfire verification, erosion analysis, forestry analysis

**Table 5:** Common sensors used in crowdsensing-based environmental monitoring systems.

Sensor Type	Main Applications
Visual Camera	real-time imaging, natural hazard detection 3D modeling and reconstruction
Microphone	acoustic noise monitoring
Wi-Fi and Bluetooth Antenna	electromagnetic pollution monitoring, spectrum sensing
Magnetometer	electric field level monitoring
Radar	surface monitoring, subsurface material mapping
Thermal Camera	heat pollution monitoring, natural gases/CO${}_{2}$ detection
Pressure/Humidity/Temperature	heat island detection, temperature monitoring
Particle Radiation	particulate radioactivity monitoring
Particulate Matter	fine particulate monitoring
Chemical Pollutants	chemical pollutant monitoring, chemical substance detection

## Data Availability

The study did not report any data.
